# Efficient Filter Design to Compensate Fabrication Imperfections in 6G Communication Systems

**DOI:** 10.3390/s23249825

**Published:** 2023-12-14

**Authors:** Ioannis Stamatopoulos, Ioannis Koutzoglou, Dimitrios I. Karatzidis, Zaharias D. Zaharis, Pavlos I. Lazaridis, Nikolaos V. Kantartzis

**Affiliations:** 1 Directorate of Transport and Communications of Eastern Thessaloniki, 54655 Thessaloniki, Greece; microf16@yahoo.co.uk; 2 School of Electrical and Computer Engineering, Aristotle University of Thessaloniki, 54124 Thessaloniki, Greece; koutzoglouioannis@gmail.com (I.K.); karatzidis@ece.auth.gr (D.I.K.); kant@auth.gr (N.V.K.); 3 School of Computing and Engineering, University of Huddersfield, Huddersfield HD1 3DH, UK; p.lazaridis@hud.ac.uk

**Keywords:** Chebyshev analysis, construction tolerances, fabrication imperfections, filters, mictrostrips, 6G wireless communications, uncertainties

## Abstract

In this paper, we present a consistent methodology for the reliable design of 6G-oriented filters with enhanced endurance to construction imperfections. The systematic formulation does not depend on the filter’s operating frequency and employs a robust strategy for obtaining new roots and poles of the filtering function. Essentially, it requires that all the local maxima of the filtering function do not fluctuate beyond the design attenuation levels for a set of predefined roots/poles distortions. To this purpose, two novel algorithms for the derivation of the appropriate filtering functions are developed, in the prior basis, together with a versatile optimization criterion and a heuristic comparison approach that guarantee optimal outcomes. Specifically, the principal idea of the first technique is to accurately extract the roots of the new polynomial from a system of equations on condition that the maximum local peaks of the distorted (due to imperfections) initial polynomial are below a prefixed threshold, such as the unit. Conversely, the second method develops an alternative polynomial, compressed in the amplitude and frequency range, so that a similar prerequisite regarding the maximum local peaks, is satisfied. It is stressed that both methods are fully generalized and may be applied to any polynomial combination, without increasing the overall complexity. The proposed framework is successfully verified in terms of theoretical examples and the numerical simulation of realistic waveguide and mictrostrip line filters, operating at frequencies from 2GHz to 65GHz, which unveil its superiority over existing schemes and implementations.

## 1. Introduction

Millimeter wave filters, such those designed for modern 6G systems [1,2,3], require meticulous standardization and high manufacturing accuracy. This is because the sensitivity of a distributed circuit increases with frequency and thus fabrication imperfections may ruin the behavior of the resulting filters in the spectrum of wireless communications [4,5,6,7]. Since the latter use printed-circuit multi-layer inserts as discontinuities in order to achieve the required performance, all the parameters and features of the substrate, such as the dielectric permittivity, the thickness, or the losses, can impact the behavior of the filter in the case of wrongly selected values [8]. Also, the inaccurate operation of the machining techniques, the milling processes, the chemical etching, the laser cutting, or the metallization methods can create several significant geometric or structural imperfections [9]. Amid them, one may discern the fluctuating dimensions, the incorrect digging depth in the substrate, the unwanted curvatures, and the wrongly positioned inserts [10]. These imperfections can seriously affect the efficiency of 6G filters by irregularly modifying their frequency response, i.e., they actually change the their transfer function. So, the bandwidth, the amplitude response, and the central frequency of the filter are likely to deviate from the initially designed ones, thus completely degrading the performance of the device. In essence, the contribution of filters in 6G systems is decisive, since their role is to separate useful signals from noise and manage interference in the frequency spectrum, thus aiding the correct data transmission [11]. Hence, any deviation in the behavior of the initially designed filtering function will immediately degrade the operation of the entire arrangement.

To evaluate the efficiency of a filter and determine potential imperfections, several key performance criteria should be satisfied. The most important are the variation of the filtering (filter transfer) function, the frequency and amplitude response of the filter, the localization of its central (operating) frequency, and its bandwidth [12]. For this purpose, current research focuses on the following areas: (a) the optimization of existing filter fabrication processes (e.g., surface roughness, metallization, and selection of proper materials), (b) the redesign of typical filter architectures according to modern standards (e.g., smooth surfaces, adaptive geometries), and (c) the development of new design strategies. The latter are deemed the most promising and are divided into two classes. The techniques of the first class assess fabrication imperfections via mathematical methods (like the one proposed herein), based on the improvement of the filtering functions, the polynomial chaos, or the enhancement of stochastic techniques [13]. The second class launches prototype manufacturing processes, such as the 3D printing, the additive manufacturing, the digital manufacturing, and the free-form fabrication [14]. These techniques build filters by selectively adding material (layer by layer), instead of subtracting, as in usual procedures.

Bearing in mind these notions and apart from the meticulous inspection of any conventional fabrication technique, modern design filter strategies try to establish standardized steps to avoid imperfections [15]. The first is the generation of the proper 3D computer-aided design (CAD ) model of the filter via the respective computer software or a reverse-engineering procedure [16]. Then, this CAD model is converted to the appropriate stereolithography format, which describes the filter as a discretized mesh, and placed with extreme accuracy in the building device to avoid staircase approximations [17]. Next, the fully automated manufacturing process starts, while some minor faults are removed via thermal treatment, sandpapering, shot-peening, surface-coating, or infiltration [18].

Recently, there has been a remarkable interest for the incorporation of emerging technologies into the compensation of filter fabrication imperfections [19,20]. The most important is the development of advanced computational packages with artificial intelligence modules to estimate the impact of potential faults or even indicate means for their correction, prior to any fabrication [21]. However, the use of thermoplastic and biogradable materials with unique properties is, also, a topic of tense research. Among such media, we can mention the acrylonitrile butadiene styrene, the PolyLactic acid, the PolyEtherEtherKetone, and various advanced ceramics [22]. Not to mention that most 3D printing techniques are revisited in order to replace inkjet schemes with material jetting, i.e., the deposition of droplets of material through moveable printer heads [23]. In addition, powder bed fusion technologies are considered to selectively fuse particles through a laser or an electron beam at specific filter areas [24]. Another promising technology for 6G filters is the 3D screen-printing process, which can design net-shaped components with complex shapes, with negligible imperfections [25]. Finally, photopolymerization platforms consisting of liquid resin have become fairly popular due the faultless filter surface finishing they attain [26]. Nonetheless, it is emphasized that the above technologies should overcome diverse challenges, before they will become widely popular. In particular, they must properly address: (a) the high cost of the fabrication equipment, (b) the constantly increasing needs for dimensional accuracy and repeatability, (c) the requirement for new materials with advanced properties, and (d) the absence of fully established fabrication standards.

A crucial feature of a filter design technique is to be fully independent of frequency. This is exactly one of the novelties of the technique proposed in this paper and achieved by directly modifying the filtering function after the systematic analysis of the detected imperfections. On the other hand, traditional approaches attempt to adapt to different frequencies by suggesting various formulations for different spectra or approximations that could offer adequate (yet not optimal) results. It becomes apparent that low sensitivity filtering functions must be employed to compensate for the frequently encountered manufacturing tolerances [27,28,29]. Considering that these functions are created by a polynomial, the Chebyshev filters (derived by the Chebyshev polynomials) exhibit the best (steepest) behavior in the out-of-band zone, yet at the expense of the highest sensitivity to mechanical defects. On the other hand, the Butterworth filters (derived by the $x^{n}$ function) are the less efficient (smoothest behavior) in the out-of-band zone, but, also, have the highest resistance to mechanical imperfections among all the filters created by polynomials of the same degree. Thus, the need for enhanced and flexible polynomials is escalating, particularly in the rapidly evolving area of 6G communications [30,31,32].

Two state-of-the-art approaches can be found in the relevant literature that, however, do not clearly associate the filter sensitivity to construction tolerances with the choice of the polynomial. Moreover, they do not promptly clarify which solution is the best before its post simulation. Nonetheless, they have the advantage of simplicity and provide the polynomial roots in a detailed way (a feature of the herein-proposed techniques as well). The first approach is the chained-function filter formulation, where the filtering function is the product of various Chebyshev and Butterworth polynomial combinations [33,34,35,36]. According to this scheme, the most efficient combination—i.e., the one that produces the steepest plot in the out-of-band zone for a given variation of the peaks in the $S_{11}$-parameter—is obtained via an appropriate criterion and a specific optimization algorithm [35]. This variation is verified through a Monte Carlo method, where the input data is the variation of the characteristic resistances of the printed circuit components. It is noteworthy to mention that the chained functions, also, consider the case of rational functions. Conversely, in the second approach, the filtering function imposes a specific pattern on the $S_{11}$-parameter, with low peaks at the edges of a slightly wider passband zone [37]. The method is validated, again, by a Monte Carlo implementation, where the input data is the variation of the coupling coefficients of the filter circuit. Principally, both of the above techniques lead to low-sensitivity filtering functions, which, as an upshot, exhibit a lower performance; an issue that requires the design of higher-order filters to retain the same performance [34].

This work addresses the impact of fabrication imperfections on the behavior of millimeter wave filters for 6G communication systems, by, again, lowering the performance of the filtering function. Nevertheless, the key difference from the aforementioned literature is that, now, the new filtering function is systematically and fully related to these manufacturing tolerances. The proposed concept stems from the roots/poles variation of the auxiliary filtering function, caused by a structural variation, which can enforce its peaks in the bandpass zone to surpass a permissible value, namely the unit in the prototype function. Then, the roots and poles are precisely relocated on the real axis, so that, in the enhanced function, the peaks decrease in magnitude and for, the same roots/poles variation, they are not larger than (the ideal case is to be equal to) unit. Such a variation should be computed in advance and this is, herein, conducted by means of the COMSOL Multiphysics^®^ simulation software [38]. Note that, throughout our analysis, the filtering function is presumed to be a real function of the frequency and, equivalently, the prototype filtering function is deemed a real function of the real variable *x*.

Based on these notions, two novel techniques for the accurate derivation of enhanced and consistent filtering functions are presented in this paper. Both algorithms are independent of the filter’s operating frequency and can be used for polynomial and ratio of polynomials (elliptic-like) filtering functions, which can lead to the straightforward and fast design of narrowband low-order millimeter filters. To ensure that the derived filtering functions are optimum (i.e., they have the steepest possible out-of-band behavior, under certain optimization constraints), an instructive criterion is introduced. Furthermore, a fully unified way of comparing the various filtering functions found in the literature with the proposed ones is described. In this manner, and although the differences among the diverse polynomials could be small, the optimum filtering function is promptly obtained.

In summary, the key novelties of the paper are: (a) the development of a generalized design methodology that does not depend on the filter’s central frequency and can effectively handle any fabrication imperfection, (b) the introduction of two precise schemes that can be directly applied to any filtering function and lead to significant enhancements, even for demanding imperfections, (c) the proposed formulation is much simpler, as it involves only the magnitude of the *S*-parameters and not the phase, and (d) the straightforward and fast extraction of the compensated filter design parameters. The new schemes are extensively validated through several real-world waveguide and microstrip line filters, with operating frequencies in the wide range between 2GHz and 65GHz, pertinent for 6G systems. Numerical results prove the advantages of the featured method, which exhibits a much better performance than existing filtering functions.

## 2. Development of the Polynomial Filtering Function Methodology

In this section, we formulate two versatile algorithms for the extraction of the necessary filtering function polynomial, significantly less vulnerable to fabrication tolerances, when the variation of its roots has, already, been estimated. According to the first method, we calculate the roots of the new polynomial from a system of equations, requiring that the maximum peaks of the distorted polynomial (i.e., its roots are varied) do not exceed a predetermined value, e.g., the unit. The resulting polynomial is shown to be optimal if it satisfies a specific optimization theorem. Conversely, the second method creates a Chebyshev polynomial, compressed in the amplitude and frequency range, so that, as above, the maximum peaks of the distorted polynomial do not exceed the unit.

### 2.1. The System-Based Optimization Method (SbOM)

Let us start from the transfer function, $S_{12}$, of a filter

(1) $$|S_{12}{\left(f\right)|}^{2}=\frac{1}{1+\varepsilon^{2}F_{n}^{2}\left(f\right)},$$

where ε is the ripple factor and $F_{n}\left(x\right)$ a Chebyshev polynomial for the case of a narrowband microwave filter. This simply implies that Equation (1) resembles an inverted Chebyshev polynomial. Bear in mind that Chebyshev polynomials have been, initially, defined as those with the smallest possible deviation from the horizontal axis among all monomial polynomials (with the unit factor at the $x^{n}$ term) of the same degree *n*. This minimum deviation is achieved by forcing all local maxima of the polynomial to be equal to each other in absolute value. In essence, Chebyshev polynomials became popular since they can produce filtering functions that are maximized outside the interval of their roots (in the prototype problem for |x|≥1), while retaining their peaks below 1, for |x|≤1 [39,40]. Therefore, they provide the steepest possible plot in the out-of-band zone from all other polynomials of the same degree. Apparently, this feature renders Chebyshev filters sensitive to fabrication imperfections, because they affect the roots of the polynomial. As Chebyshev polynomials exhibit the smallest possible deviation from the horizontal axis, a change of their roots causes a definite increase of, at least, one local maximum (peak) beyond 1. This means that the filtering function does not satisfy the typical design standards, e.g., it leads to $|S_{12}|<-0.043\;$dB or $|S_{11}|>-20\;$dB, for ε=0.1. On the other hand, the roots in Butterworth filters and thus the local maxima are all at 0; so, a change of the roots does create peaks, yet there is enough space for them to remain below the maximum level of variation.

In the present work, we consider that the seeking polynomial, $F_{n}\left(x\right)$, has real roots in [−1,1] (i.e., arranged as $-1\leq x_{1}\leq \cdots \leq x_{n}\leq 1$), its peaks in [−1,1] do not exceed 1, and $F_{n}\left(x\right)$ crosses the (1,1) point. Hence, $F_{n}\left(x\right)$ is given by

(2) $$F_{n}\left(x\right)=K\prod_{i=1}^{n}\left(x-x_{i}\right),\;\;\mathrm{where}\;K=1/\left[\prod_{i=1}^{n}\left(1-x_{i}\right)\right]$$

or $F_{n}\left(x\right)$ crosses the (1,1) point. For even symmetry, $F_{n}\left(x\right)$ can be written as

(3) $$F_{n}\left(x\right)=K\prod_{i=1}^{n/2}\left(x^{2}-x_{i}^{2}\right),\;\;\mathrm{where}\;K=1/\left[\prod_{i=1}^{n/2}\left(1-x_{i}^{2}\right)\right],$$

with $0\leq x_{1}\leq \cdots \leq x_{n/2}\leq 1$. Owing to mechanical imperfections, the roots of the polynomial become $x_{i}\pm d_{i}$, with $d_{i}\geq 0$ the maximum variance of the *i*th root. For simplicity, we assume that the increment and decrement of the *i*th root are equal, while, below, this constraint can be relaxed. Obviously, the total number of $x_{i}\pm d_{i}$ combinations of the roots produces a large set of distorted polynomials whose local peaks vary in size. A local peak receives its maximum value for a specific combination. Hence, to find the proper $x_{i}$ roots, we require that all these maxima should not to exceed 1 in the prototype problem. This yields a system of n−1 equations, half of which are used to set the maximum peaks to 1 and half to acquire their abscissas. It is emphasized that in the case of even symmetry, the number of equations for the maximum peaks is n/2 and for the abscissas (n/2)−1. Conversely, in the case of odd symmetry, these numbers are (n−1)/2 for both the peaks and the abscissas. Furthermore, it will be numerically shown in the following paragraphs that, via an optimization criterion, the closest each local maximum peak is located to 1, the steeper the plot of the resulting polynomial is produced outside [−1,1].

Prior to formulating the new SbOM, we focus on some important characteristics of $F_{n}\left(x\right)$, considering that the arrangement of its $-1\leq x_{1}\leq \cdots \leq x_{n}\leq 1$ roots is maintained. Specifically, we provide the subsequent propositions:

**Proposition** **1.** *Denoting as i the peak between the $x_{i}$ and $x_{i+1}$ root, it is stated that this peak increases if $x_{i}$ decreases (similarly, if the $x_{i+1}$ root increases). Indeed, if the abscissa point of the ith peak is denoted as $q_{i}$, then the distorted polynomial, $G_{n}\left(x,d_{i}\right)$, at $q_{i}$, can be defined as*

$$|G_{n}\left(q_{i},d_{i})\right|\equiv {\left(-1\right)}^{i}K\left[q_{i}-\left(x_{i}-d_{i}\right)\right]\prod_{k=1,\neq i}^{n}\left(q_{i}-x_{k}\right)$$

*and as $x_{i}<q_{i}$, it holds that*

$$\begin{array}{cc} |G_{n}\left(q_{i},d_{i})\right|={\left(-1\right)}^{i}K\left[q_{i}-\left(x_{i}-d_{i}\right)\right] & \prod_{k=1,\neq i}^{n}\left(q_{i}-x_{k}\right)\\ \; & >{\left(-1\right)}^{i}K\left(q_{i}-x_{i}\right)\prod_{k=1,\neq i}^{n}\left(q_{i}-x_{k}\right)=\left|F_{n}\left(q_{i}\right)\right|. \end{array}$$

*Since the prior inequality is valid for $q_{i}$, it is, also, valid for the new $\xi_{i}$ points that are the abscissas of the $G_{n}\left(x,d_{i}\right)$ peaks; a fact which proves the proposition.*

**Proposition** **2.** *For a $d_{i}$ small relative to the $x_{i}$ root, the maximum of the ith peak occurs when the $x_{1}\cdots x_{i}$ roots depart from the $x_{i+1}\cdots x_{n}$ ones. The reasoning is the same as the one used to compare the ith maximum of the $F_{n}\left(x\right)$ with that of the $G_{n}\left(q_{i},d_{i}\right)$ above. It is simply, now, considered that the roots move consecutively (i.e., one at a time). Hence, the maximum of the ith peak occurs for the $x_{1}-d_{1},\cdots ,x_{i}-d_{i},x_{i+1}+d_{i+1},\cdots ,x_{n}+d_{n}$ combination of the roots.*

**Proposition** **3.** *The absolute value of every $F_{n}\left(x\right)$ peak is smaller than *1*, since its maximum is not larger than *1*.*

**Proposition** **4.** *The abscissas of all roots will be in [−1,1], due to the fact that $F_{n}\left(x\right)$ crosses the (1,1) point. Indeed, if there were an $F_{n}\left(x\right)$ root outside [−1,1], then the (1,1) point would act as a peak between two roots, implying that there would exist a combination of the roots for which the specific peak would be larger than *1*; i.e., a complete contradiction to the way the roots are calculated.*

**Proposition** **5.** *The previously defined coefficient K is always smaller than its $K_{\mathrm{Chebyshev}}$ counterpart of the Chebyshev polynomial. This is because, in terms of Proposition 3, the absolute values of the $F_{n}\left(x\right)$ peaks are smaller than *1* and thus the peaks of the monic $F_{n}\left(x\right)/K$ polynomial are below the level of the corresponding monic Chebyshev polynomial. Since the latter has the smallest possible deviation from the horizontal axis (and is unique), the point where $|F_{n}\left(x\right)/K|$ crosses x=1 is higher than the maximum of the monic Chebyshev polynomial, $C_{n}\left(x\right)$, and less than *1*. This implies that $|C_{n}\left(1\right)|=1/|K_{\mathrm{Chebyshev}}\left|<\right|F_{n}\left(1\right)/K|=1/|K|<1$, which proves the proposition.*

It can be numerically verified that |K| has its maximum value for the optimum polynomial, as explained in the next paragraphs. Similarly, we can derive that the larger the root variation, the more the new filtering function resembles a Butterworth function. Moreover, in the worst-case scenario, it is presumed that the roots fluctuate independently to each other, which may produce less steep filtering functions. However, the relation between fluctuations depends on the filter implementation.

#### 2.1.1. Calculation of Maxima

In general, if $d_{i}$ are large compared with the roots, then we have to take into account many variations of root combinations to retrieve every maximum local peak of the polynomial. Nonetheless, for small $d_{i}$, we may consider that the arrangement of $x_{i}$ remains and, by means of Proposition 2, obtain the *i*th maximum of $F_{n}\left(x\right)$. On the other hand, if $d_{i}$ are large compared with the distance between the roots or some of the roots coincide, then the auxiliary arrangement of $x_{i}$ can no longer remain and movements of roots along different directions may provide the local maximum peaks. To overcome this hindrance, the algorithm we employ controls the values of the local peaks along every root movement. Actually, if there are doubts for the maxima of a local peak, then other variations of root combinations can be used. Nevertheless, this happens only for a few cases in our analysis.

To extract $x_{i}$, let us, now, consider that the increment, $d_{i}^{+}$, and the decrement, $d_{i}^{-}$, of the *i*th root differ. According to the preceding notions, for relatively small root variations, the maximum of the local *i*th peak occurs for the $x_{1}-d_{1}^{-},\ldots ,x_{i}-d_{i}^{-},x_{i+1}+d_{i+1}^{+},\ldots ,x_{n}+d_{n}^{+}$ roots and the polynomial $F_{n}^{\left(i\right)}\left(x\right)$ (called distorted polynomial) is given by

(4) $$F_{n}^{\left(i\right)}\left(x\right)\equiv K\prod_{k=1}^{i}\left(x-x_{k}+d_{k}^{-}\right)\prod_{k=i+1}^{n}\left(x-x_{k}-d_{k}^{+}\right).$$

For the case of even symmetry, the corresponding root variation is

(5) $$-x_{n/2}-d_{n/2}^{+},\ldots ,-x_{1}-d_{1}^{+},x_{1}-d_{1}^{-},\ldots ,x_{i}-d_{i}^{-},x_{i+1}+d_{i+1}^{+},\ldots ,x_{n/2}+d_{n/2}^{+},$$

while the distorted polynomial reads

(6) $$\begin{array}{c} F_{n}^{\left(i\right)}\left(x\right)\;\equiv \;K\prod_{k=1}^{i}\left\{\left[{\left(x\;+\;d_{k}^{-}\right)}^{2}\;-\;x_{k}^{2}\right]\;+\;\left(d_{k}^{+}\;-\;d_{k}^{-}\right)\left(x\;-\;x_{k}\;+\;d_{k}^{-}\right)\right\}\prod_{k=i+1}^{n/2}\left[x^{2}\;-\;{\left(x_{k}\;+\;d_{k}^{+}\right)}^{2}\right], \end{array}$$

with 1≤i≤(n/2)−1, and

(7) $$F_{n}^{\left(0\right)}\left(x\right)\equiv K\prod_{k=1}^{n/2}\left[x^{2}-{\left(x_{k}+d_{k}^{+}\right)}^{2}\right],$$

for i=0 (the distorted polynomial with maximum local peak between the $x_{1}-d_{1}^{+},x_{1}+d_{1}^{+}$) and *K* as defined in Equation (3).

In this context, for the required $x_{i}$, we must solve the next system of n−1 equations

(8) $$\begin{array}{cc} \left|\max_{i}F_{n}^{\left(i\right)}\left(x\right)\right| & =\left|F_{n}^{\left(i\right)}\left(\xi_{i}\right)\right|={\left(-1\right)}^{(n/2)-i}F_{n}^{\left(i\right)}\left(\xi_{i}\right)=p_{i}\\ \; & \Rightarrow \;{\left(-1\right)}^{(n/2)-i}K\prod_{k=1}^{i}\left\{\left[{\left(\xi_{i}\;+\;d_{k}^{-}\right)}^{2}\;-\;x_{k}^{2}\right]\;+\;\left(d_{k}^{+}\;-\;d_{k}^{-}\right)\left(\xi_{i}\;-\;x_{k}\;+\;d_{k}^{-}\right)\right\}\\ \; & \;\times \prod_{k=i+1}^{n/2}\left[\xi_{i}^{2}\;-\;{\left(x_{k}\;+\;d_{k}^{+}\right)}^{2}\right]\;=\;p_{i}, \end{array}$$

for 1≤i≤(n/2)−1, and

(9) $$\begin{array}{c} \left|\max_{i=0}F_{n}^{\left(0\right)}\left(x\right)\right|=\left|F_{n}^{\left(0\right)}\left(0\right)\right|={\left(-1\right)}^{n/2}F_{n}^{\left(0\right)}\left(0\right)=p_{0}\Rightarrow K\prod_{k=1}^{n/2}{\left(x_{k}+d_{k}^{+}\right)}^{2}=p_{0}, \end{array}$$

for i=0, and

(10) $$\begin{array}{cc} {\left.\frac{\partial F_{n}^{\left(i\right)}\left(x\right)}{\partial x}\right|}_{x=\xi_{i}}=0 & \Rightarrow \sum_{r=1}^{i}\{\left[2\left(\xi_{i}\;+\;d_{r}^{-}\right)\;+\;\left(d_{r}^{+}\;-\;d_{r}^{-}\right)\right]\\ \; & \;\times \;\;\left.\prod_{k=1,\neq r}^{i}\left\{\left[{\left(\xi_{i}\;+\;d_{k}^{-}\right)}^{2}-x_{k}^{2}\right]+\left(d_{k}^{+}\;-\;d_{k}^{-}\right)\left(\xi_{i}\;-\;x_{k}+d_{k}^{-}\right)\right\}\prod_{k=i+1}^{n/2}\left[\xi_{i}^{2}\;-\;{\left(x_{k}\;+\;d_{k}^{+}\right)}^{2}\right]\;\right\}\\ \; & \;+\;\;\sum_{r=i+1}^{n/2}\;\left\{2\xi_{i}\prod_{k=1}^{i}\left\{\left[{\left(\xi_{i}\;+\;d_{k}^{-}\right)}^{2}\;-\;x_{k}^{2}\right]\;+\;\left(d_{k}^{+}\;-\;d_{k}^{-}\right)\left(\xi_{i}\;-\;x_{k}\;+\;d_{k}^{-}\right)\;\right\}\;\;\prod_{k=i+1,\neq r}^{n/2}\left[(\xi_{i}^{2}\;-\;{\left(x_{k}\;+\;d_{k}^{+}\right)}^{2}\right]\;\right\}\;=\;0, \end{array}$$

for 1≤i≤(n/2)−1. In Equations (8) and (10), $\xi_{i}$ is a point where the partial derivative of $F_{n}^{\left(i\right)}\left(x\right)$ with respect to *x* is 0, for $\xi_{0}=0$. Moreover, the $|\max_{i}F_{n}^{\left(i\right)}\left(x\right)|$ term in Equations (8) and (9) is the value of the maximum local *i*th peak of the $F_{n}^{\left(i\right)}\left(x\right)$ polynomial that appears at point $\xi_{i}$ between the $x_{i}-d_{i}^{-}$ and $x_{i+1}+d_{i+1}^{+}$ roots of $F_{n}^{\left(i\right)}\left(x\right)$.

Conversely, for the case of odd symmetry (i.e., $x_{1}=0$), we consider that $d_{1}^{-}=d_{1}^{+}=d_{1}$ and the respective root variation becomes

(11) $$\begin{array}{c} -x_{(n+1)/2}\;-\;d_{(n+1)/2}^{+},\ldots ,x_{1}\;-\;d_{1}^{-},\ldots ,x_{i}\;-\;d_{i}^{-},x_{i+1}\;+\;d_{i+1}^{+},\ldots ,x_{(n+1)/2}\;+\;d_{(n+1)/2}^{+}, \end{array}$$

whereas the distorted polynomial is given by

(12) $$\begin{array}{c} F_{n}^{\left(i\right)}\;\left(x\right)\;\equiv \;K\left(x\;+\;d_{1}\right)\;\prod_{k=2}^{i}\;\left\{\;\left[\;{\left(x\;+\;d_{k}^{-}\right)}^{2}\;\;-\;x_{k}^{2}\right]\;\;+\;\left(d_{k}^{+}\;-\;d_{k}^{-}\right)\left(x\;-\;x_{k}\;+\;d_{k}^{-}\right)\;\right\}\;\;\;\prod_{k=i+1}^{(n+1)/2}\left[x^{2}\;-\;{\left(x_{k}\;+\;d_{k}^{+}\right)}^{2}\right] \end{array}$$

for 2≤i≤(n+1)/2−1, and

(13) $$\begin{array}{c} F_{n}^{\left(1\right)}\left(x\right)\equiv K\left(x+d_{1}\right)\prod_{k=2}^{(n+1)/2}\left[x^{2}-{\left(x_{k}+d_{k}^{+}\right)}^{2}\right], \end{array}$$

for i=1, where $K=1/\left[\prod_{i=2}^{(n+1)/2}\left(1-x_{i}^{2}\right)\right]$. Thus, Equations (8)–(10) can, now, be written as

(14) $$\begin{array}{cc} \left|\max_{i}F_{n}^{\left(i\right)}\left(x\right)\right| & =\left|F_{n}^{\left(i\right)}\left(\xi_{i}\right)\right|={\left(-1\right)}^{[(n+1)/2]-i}F_{n}^{\left(i\right)}\left(\xi_{i}\right)=p_{i}\\ \; & \Rightarrow {\left(-1\right)}^{[(n+1)/2]-i}K\left(\xi_{i}\;+\;d_{1}\right)\prod_{k=2}^{i}\left\{\;\left[{\left(\xi_{i}\;+\;d_{k}^{-}\right)}^{2}\;-\;x_{k}^{2}\right]\;+\;\left(d_{k}^{+}\;-\;d_{k}^{-}\right)\left(\xi_{i}\;-\;x_{k}\;+\;d_{k}^{-}\right)\right\}\\ \; & \;\times \prod_{k=i+1}^{(n+1)/2}\left[\xi_{i}^{2}-{\left(x_{k}+d_{k}^{+}\right)}^{2}\right]=p_{i}, \end{array}$$

for 2≤i≤(n+1)/2−1, and

(15) $$\begin{array}{c} \left|\max_{i=1}F_{n}^{\left(1\right)}\left(x\right)\right|=\left|F_{n}^{\left(1\right)}\left(1\right)\right|=p_{1}\Rightarrow K\left(\xi_{1}+d_{1}\right)\prod_{k=2}^{(n+1)/2}\left[{\left(x_{k}+d_{k}^{+}\right)}^{2}-\xi_{1}^{2}\right]=p_{1}, \end{array}$$

for i=1, and

(16) $$\begin{array}{cc} \; & {\left.\frac{\partial F_{n}^{\left(i\right)}\left(x\right)}{\partial x}\right|}_{x=\xi_{i}}=0\Rightarrow \prod_{k=2}^{i}\left\{\left[{\left(\xi_{i}+d_{k}^{-}\right)}^{2}-x_{k}^{2}\right]+\left(d_{k}^{+}-d_{k}^{-}\right)\left(\xi_{i}-x_{k}+d_{k}^{-}\right)\right\}\prod_{k=i+1}^{(n+1)/2}\left[\xi_{i}^{2}-{\left(x_{k}+d_{k}^{+}\right)}^{2}\right]\\ \; & \;+\;\sum_{r=2}^{i}\left\{\;\left[2\left(\xi_{i}\;+\;d_{r}^{-}\right)\;+\;\left(d_{r}^{+}\;-\;d_{r}^{-}\right)\right]\left(\xi_{i}\;+\;d_{1}\right)\;\prod_{k=2,\neq r}^{i}\;\left\{\;\left[{\left(\xi_{i}\;+\;d_{k}^{-}\right)}^{2}\;-\;x_{k}^{2}\right]\;+\;\left(d_{k}^{+}\;-\;d_{k}^{-}\right)\left(\xi_{i}\;-\;x_{k}\;+\;d_{k}^{-}\right)\right\}\;\;\prod_{k=i+1}^{(n+1)/2}\left[\xi_{i}^{2}\;-\;{\left(x_{k}\;+\;d_{k}^{+}\right)}^{2}\right\}\;\;\right\}\\ \; & \;+\;\sum_{r=i+1}^{(n+1)/2}\left\{2\xi_{i}\left(\xi_{i}\;+\;d_{1}\right)\;\prod_{k=2}^{i}\left\{\;\left[{\left(\xi_{i}\;+\;d_{k}^{-}\right)}^{2}\;-\;x_{k}^{2}\right]\;+\;\left(d_{k}^{+}\;-\;d_{k}^{-}\right)\left(\xi_{i}\;-\;x_{k}\;+\;d_{k}^{-}\right)\right\}\;\prod_{k=i+1,\neq r}^{(n+1)/2}\left[\xi_{i}^{2}\;-\;{\left(x_{k}\;+\;d_{k}^{+}\right)}^{2}\right]\;\right\}=0, \end{array}$$

for 2≤i≤(n+1)/2−1, and

(17) $$\begin{array}{c} \prod_{k=2}^{(n+1)/2}\left[\xi_{1}^{2}-{\left(x_{k}+d_{k}^{+}\right)}^{2}\right]+2\xi_{1}\left(\xi_{1}+d_{1}\right)\sum_{r=2}^{(n+1)/2}\prod_{k=2,\neq r}^{(n+1)/2}\left[\xi_{1}^{2}-{\left(x_{k}+d_{k}^{+}\right)}^{2}\right]=0, \end{array}$$

for i=1.

Notice that in Equations (8), (14), and (15), we have $p_{i}\leq 1$. The reason for not imposing $p_{i}=1$ is that, generally, the system of Equations (8)–(10)—or, equivalently, the system of Equations (14)–(17)—is not solvable for every $p_{i}=1$. Hence, in our algorithm, we, initially, set all $p_{i}=1$ and, in the end, some roots may be found equal to each other, implying that for some $p_{i}$ (whose calculation is not required) it holds that $p_{i}<1$.

#### 2.1.2. Optimal Formulation

A critical aspect in the development of the proposed SbOM is to investigate whether the solution of Equations (8)–(10) is optimal, namely if it provides the maximum possible value of $|F_{n}\left(x\right)|$, for |x|≥1. This is the case if the solution satisfies a specific optimization theorem. Actually, there are some proofs in the literature concerning the behavior of Chebyshev polynomials outside [−1,1]. Some of them are based on the alternation of the pronoun of the difference between the Chebyshev polynomial and another polynomial function that intersects it [39]. Others rely on the representation of the Chebyshev polynomial as a Lagrange interpolation [40]. Such approaches, however, can not be applied to the proposed technique, since our objective is to draw a conclusion about the behavior of $F_{n}\left(x\right)$ by observing $F_{n}^{\left(i\right)}\left(x\right)$, which are different polynomials. Furthermore, to maximize $|F_{n}\left(x_{0}\right)|$, for $|x_{0}|\geq 1$, when $\max|F_{n}\left(x\right)|\leq 1$, for |x|≤1, is equivalent to minimize $\max|F_{n}\left(x\right)|$, for |x|≤1, when $|F_{n}\left(x_{0}\right)|=1$, for some random $|x_{0}|\geq 1$, i.e., a linear relationship between the roots [41]. In this framework, our optimization problem opts for the minimization of $|\max_{i}F_{n}^{\left(i\right)}\left(x\right)|$, for |x|≤1, when $|F_{n}\left(x_{0}\right)|=1$, for some random $|x_{0}|\geq 1$, as, also, alternatively described in [42].

Focusing on our approach, we firstly obtain a solution from (8)–(10) and then examine if this is optimal, according to whether it satisfies a specific optimization theorem. Explicitly, we want to *optimize the polynomial function $F_{n}\left(x_{0}\right)$, for $x_{0}>1$, whose variables are its roots $x_{i}\;\left(1\leq i\leq n/2\right)$, under the constraints $|\max_{i}F_{n}^{\left(i\right)}\left(x\right)|\leq p_{i}$, with $p_{i}\leq 1$*. In essence, the formulation, introduced herein, can be deemed as an appropriately tailored version of the optimization theorem of a constrained function [43], which uses the Lagrange λ multipliers. Our aim is to prove that $\lambda_{i}>0$, when the $x_{i}$ are such that $|\max_{i}F_{n}^{\left(i\right)}\left(x\right)|=p_{i}$ are valid. Differently speaking, $F_{n}\left(x_{0}\right)$ will be maximized, when $|\max_{i}F_{n}^{\left(i\right)}\left(x\right)|=p_{i}$ apply and therefore $F_{n}\left(x_{0}\right)$ will be larger than the value of any other polynomial at the same point $x_{0}$, whose local maxima are not larger than $p_{i}$ and at least one is smaller. Hence, we launch the representation of

(18) $$\begin{array}{c} L\left(x_{1},x_{2},\ldots ,x_{n/2}\right)\equiv F_{n}\left(x_{0},x_{1},\ldots ,x_{n/2}\right)-\sum_{i=1}^{n/2}\lambda_{i}\left(\left|\max_{i}F_{n}^{\left(i\right)}\left(x\right)\right|-p_{i}\right)=0 \end{array}$$

and the system of equations $\partial L\left(x_{1},x_{2},\ldots ,x_{n/2}\right)/\partial x_{i}=0$, for 1≤i≤n/2. Then, owing to Equations (8)–(10), we derive the following system in matrix form

(19) $$\begin{array}{c} {}[M]\cdot [\Lambda ]=[\Gamma ], \end{array}$$

where

$$\begin{array}{cc} \; & \left[\Lambda \right]={\left[\lambda_{1},\ldots ,\lambda_{n/2}\right]}^{T},\\ \; & \left[\Gamma \right]=F_{n}\left(x_{0}\right)\left(1-x_{0}^{2}\right){\left[\frac{1}{1-x_{1}^{2}},\ldots ,\frac{1}{1-x_{n/2}^{2}}\right]}^{T},\\ \; & \left[M\right]=\left[m_{i,j}\right]=\left\{\begin{array}{cc} {\left(-1\right)}^{(n/2)+1-i}p_{i}\frac{\left(x_{i}+d_{i}\right)\left[\left(1/x_{i}\right)+d_{i}\right]-\xi_{j}^{2}}{{\left(x_{i}+d_{i}\right)}^{2}-\xi_{j}^{2}}, & \mathrm{for}\;\;j\leq i\\ {\left(-1\right)}^{(n/2)+1-i}p_{i}\frac{1-{\left(\xi_{j}+d_{i}\right)}^{2}}{x_{i}^{2}-{\left(\xi_{j}+d_{i}\right)}^{2}}, & \mathrm{for}\;\;j>i \end{array}\right. \end{array}$$

Consequently, when the above theorem is satisfied, namely when $\lambda_{i}>0$, we can reliably draw the subsequent significant deductions, whose validity does not depend (by any means) on the filter’s operating frequency:

- The polynomial in the out-of-band zone, i.e., for |x|≥1, is maximized when the maxima of the local peaks receive the values of $p_{i}$ and is not smaller than them.
- The polynomial in the out-of-band zone, i.e., for |x|≥1, is better than any other polynomial, whose local maxima are not larger than $p_{i}$ and at least one of them is smaller.
- Since $p_{i}$ can be defined arbitrarily, we can set all of them equal to 1, on the condition that Equations (8)–(10) are solvable for this selection. This is the maximum normalized value and provides the maximum possible polynomial value for |x|≥1, namely the steepest possible plot in the out-of-band zone.
- When all the maximum peaks $p_{i}$ of a polynomial are one-to-one smaller than or equal to the maximum peaks $p_{i}^{\prime }$ of another polynomial and at least one $p_{i}$ is less than one $p_{i}^{\prime }$, then the former polynomial is less steep than the latter in the out-of-band zone.

As presented in the next sections, we successfully apply the prior theorem to various solutions of the Equations (8)–(10) system, for different $d_{i}$ combinations during the design of sixth-order filters. Results reveal that, in all cases, the $\lambda_{i}$s are found to be positive and therefore our solutions are, indeed, the optimal ones. Moreover, since $x_{i}$ and $\xi_{i}$ are known analytically, it is possible that the above theorem can provide an alternative interpretation to the behavior of Chebyshev polynomials outside [−1,1]. It is stressed that the specific procedure may be used to demonstrate the advantages of the proposed filtering function over existing options, namely it can precisely produce the steepest possible out-of-band plot. Finally, we can deduce that the SbOM leads to the optimum polynomial when the $d_{i}$s are none-zero, whereas the Chebyshev polynomial is the optimum one when the $d_{i}$s vanish.

#### 2.1.3. Solution of the Equation System

Depending on the symmetry type, we solve sequentially the previously derived system of Equations: (a) (8)–(10) for $x_{1},\xi_{2},x_{2},\xi_{3},x_{3},\ldots ,\xi_{n/2},x_{n/2},x_{1},\ldots$ (even symmetry) or (b) (14)–(17) for $x_{2},\xi_{2},x_{3},\ldots ,\xi_{(n-1)/2},x_{(n+1)/2},\xi_{1},x_{2},\ldots$ (odd symmetry), until the full convergence of the roots. Recall that $\xi_{i}$ are the abscissas of the maximum local peaks, while our guess values are the roots of the Chebyshev polynomial of the same degree, each time taking into account that $p_{i}=1$. To this goal, our algorithm entails that if a $x_{i}$ is calculated larger than the $x_{i+1}$, then $x_{i}$ receives the value of $x_{i+1}$ (double root), whereas if a $x_{i}$ is calculated smaller than the $x_{i-1}$, then $x_{i-1}$ receives the value of $x_{i}$ (double root). The former case results in $p_{i}<1$ and the latter in $p_{i-1}<1$, although we do not have to compute them. Observe that for the system of Equations (8)–(10), there is no solution when, finally, $x_{1}<0$ (even symmetry) or for the system of Equations (14)–(17), when $x_{2}<0$ (odd symmetry). In the case that the final solution has double roots, it should be checked whether it satisfies other variations of root combinations. Attention must be, also, drawn to the fact that when not all roots are single, the solution is not unique and thus one can not possibly deduce which of them is the optimum, unless their plots are a posteriori examined.

**Figure 1:**
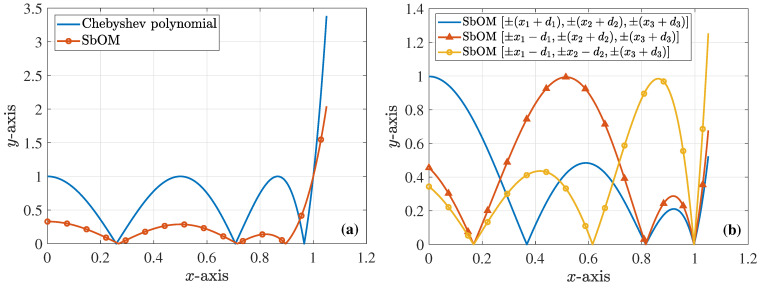
Diverse sixth-order polynomial realizations for the design of the corresponding filter (absolute values are depicted). (**a**) The Chebyshev polynomial and the polynomial derived via the proposed SbOM, with $d_{1}=d_{2}=d_{3}=0.1$. (**b**) Three different polynomials, derived via the proposed SbOM (with $d_{1}=d_{2}=d_{3}=0.1$) as this is distorted by various combinations of the roots. The first, second, and third maximum peak, of each case, respectively, do not exceed 1.

**Figure 2:**
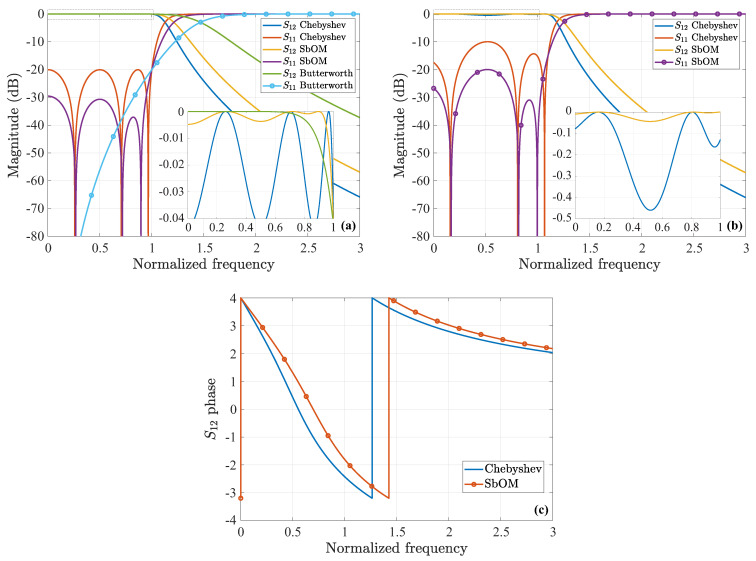
Magnitude of the *S*-parameters (ε=0.1) for the sixth-order filter, extracted via (**a**) the Chebyshev, Butterworth, and SbOM filtering functions of Figure 1a and (**b**) the Chebyshev and SbOM (Figure 1b) filtering functions, distorted by the $\pm \left(x_{1}+d_{1}\right),\pm \left(x_{2}+d_{2}\right),\pm \left(x_{3}+d_{3}\right)$ combination of the roots. (**c**) Phase of the $S_{12}$-parameters for the distorted filtering functions of (**b**). The inlet plots in (**a,b**) present a magnification of the dashed region.

As a first example to comprehend the prior scheme, consider a sixth-order filter with $d_{1}=d_{2}=d_{3}=0.1\;\left(d_{i}^{+}=d_{i}^{-}\right)$. Applying the SbOM, we find that $x_{1}=0.268,x_{2}=0.715,x_{3}=0.896$, while for the corresponding Chebyshev polynomial the roots are $x_{1}=0.2588,x_{2}=0.7071,x_{3}=0.9659$. Let us, now, more elaborately outline the initial steps of our algorithm. Focusing on the even symmetry case, we employ Equation (9) to acquire $x_{1}$ (while $x_{2}$ and $x_{3}$ come from the Chebyshev polynomial), namely

$$\begin{array}{c} \frac{{\left(x_{1}+0.1\right)}^{2}{\left(0.7071+0.1\right)}^{2}{\left(0.9659+0.1\right)}^{2}}{\left(1-x_{1}^{2}\right)\left(1-0.7071^{2}\right)\left(1-0.9659^{2}\right)}=1\Rightarrow x_{1}=0.1115, \end{array}$$

where we selected the positive value. Then, this $x_{1}$ is plugged into Equation (10) to compute $\xi_{1}$ (again, $x_{2}$ and $x_{3}$ are obtained from the Chebyshev polynomial) as

$$\begin{array}{cc} (\xi_{1} & +0.1)\left[\xi_{1}^{2}-{\left(0.7071+0.1\right)}^{2}\right]\left[\xi_{1}^{2}-{\left(0.9659+0.1\right)}^{2}\right]\\ \; & +\xi_{1}\left[{\left(\xi_{1}+0.1\right)}^{2}-0.1115^{2}\right]\left[\xi_{1}^{2}-{\left(0.9659+0.1\right)}^{2}\right]\\ \; & +\xi_{1}\left[{\left(\xi_{1}+0.1\right)}^{2}-0.1115^{2}\right]\left[\xi_{1}^{2}-{\left(0.7071+0.1\right)}^{2}\right]=0\Rightarrow \xi_{1}=0.4919, \end{array}$$

where we chose the solution between $x_{1}$ and $x_{2}$. Next, this $\xi_{1}$ is substituted into Equation (8) to extract $x_{2}$ (now, $x_{1}$ is acquired from the previous step and $x_{3}$ from the Chebyshev polynomial), i.e.,

$$\begin{array}{c} \frac{\left[{\left(0.4919+0.1\right)}^{2}\;-\;0.1115^{2}\right]\;\left[0.4919^{2}\;-\;{\left(x_{2}\;+\;0.1\right)}^{2}\right]\;\left[0.4919^{2}\;-\;{\left(0.9659+0.1\right)}^{2}\right]}{\left(1-0.1115^{2}\right)\left(1-x_{2}^{2}\right)\left(1-0.9659^{2}\right)}\;=\;1\;\Rightarrow \;x_{2}\;=\;0.5317, \end{array}$$

which is, again, the positive value. Similarly, this $x_{2}$ is plugged into Equation (10) to obtain $\xi_{2}$ (with $x_{1}$ obtained from the previous step and $x_{3}$ from the Chebyshev polynomial) and so on until the convergence of the solution. The plot of the new polynomial along with its typical Chebyshev counterpart are illustrated in Figure 1a. Additionally, Figure 1b presents three polynomial implementations, extracted through the SbOM as this is distorted by diverse combinations of the roots, where none of the maximum local peaks is larger than 1. In order to further elaborate with our analysis, Figure 2a shows the magnitude of the *S*-parameters for our sixth-order filter, derived though the Chebyshev, Butterworth, and SbOM filtering functions of Figure 1a. Observe that the SbOM results lie between the Chebyshev and Butterworth ones, while the $|S_{11}|$ obtained via the SbOM is at least 10dB lower than its Chebyshev counterpart. On the other hand, Figure 2b compares the magnitude of the *S*-parameters for the sixth-order filter, retrieved through the Chebyshev and SbOM filtering functions that are distorted by the $\pm \left(x_{1}+d_{1}\right),\pm \left(x_{2}+d_{2}\right),\pm \left(x_{3}+d_{3}\right)$ combination of the roots. It can be, clearly, detected that the $|S_{11}|$ acquired via the distorted SbOM never exceeds the level of −20dB (complying fully with our design requirements), unlike the distorted Chebyshev outcome. The latter deductions are, also, verified by Figure 2c which indicates a definite lag in the phase of the $S_{12}$-parameters, acquired from the distorted Chebyshev and SbOM filtering functions.

**Figure 3:**
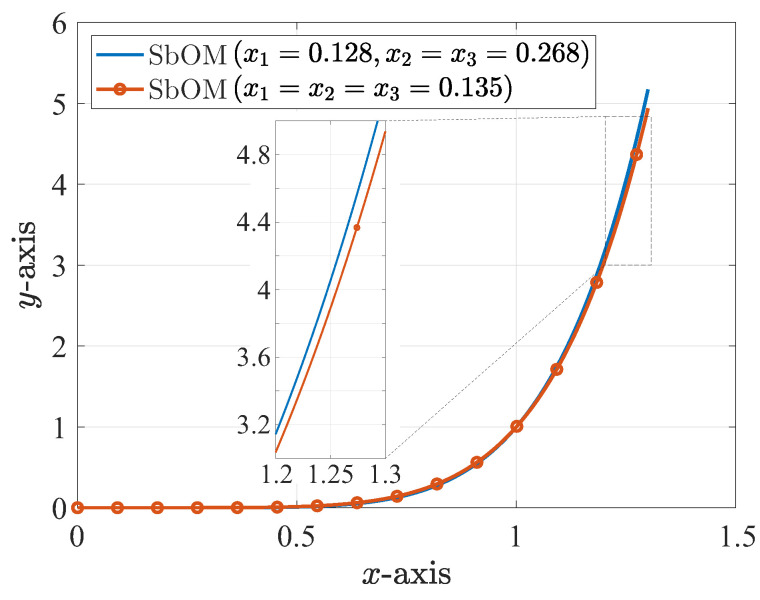
Two sixth-order polynomial realizations of the same kind for the design of the corresponding sixth-order filter, derived via the proposed SbOM for two sets of roots, with $d_{1}=0.9,d_{2}=0.2,d_{3}=0.7$ (absolute values are depicted). The inlet plot presents a magnification of the dashed region.

Secondly, we examine a sixth-order filter with $d_{1}=0.9,d_{2}=0.2,d_{3}=0.7\;\left(d_{i}^{+}=d_{i}^{-}\right)$. The SbOM leads to $x_{1}=x_{2}=x_{3}=0.26$. This is not an acceptable outcome because the third maximum peak (abscissa closest to 1) is larger than 1, for the distortion described by the $\pm \left(x_{1}+0.9\right),\pm x_{2}-0.2,\pm x_{3}-0.7$ combination of the roots (i.e., $K\left[x^{2}-{\left(x_{1}+0.9\right)}^{2}\right]$$\left[{\left(x+0.2\right)}^{2}-x_{2}^{2}\right]\left[{\left(x+0.7\right)}^{2}-x_{3}^{2}\right]$). Note that this combination does not satisfy the rule of Proposition 2. In contrast, a feasible solution is the set of $x_{1}=0.128,x_{2}=x_{3}=0.268$. Therefore, the third maximum peak is equal to 1, for the distortions denoted by the $\pm \left(x_{1}+0.9\right),\pm x_{2}-0.2,\pm x_{3}-0.7$ and $\pm x_{1}-0.9,\pm x_{2}-0.2,\pm \left(x_{3}+0.7\right)$ combinations of the roots (namely, $K\left[x^{2}-{\left(x_{1}+0.9\right)}^{2}\right]\left[{\left(x+0.2\right)}^{2}-x_{2}^{2}\right]\left[{\left(x+0.7\right)}^{2}-x_{3}^{2}\right]$ as well as $K\left[{\left(x+0.9\right)}^{2}-x_{1}^{2}\right]\left[{\left(x+0.2\right)}^{2}-x_{2}^{2}\right]\left[x^{2}-{\left(x_{3}+0.7\right)}^{2}\right]$) and the maxima of the other peaks are smaller than 1. Nevertheless, this solution is not unique, since the $x_{1}=x_{2}=x_{3}=0.135$ set is, also, acceptable. Thus, the third maximum peak is equal to 1, for the distortion described by the $\pm \left(x_{1}+0.9\right),\pm x_{2}-0.2,\pm x_{3}-0.7$ combination of the roots and the maxima of the other peaks are smaller than 1 as well as smaller than those of the first solution. This reveals that the $p_{i}$ of the latter solution are smaller than those of the former one, which is, finally, preferred. Our selection is, also, verified by means of Figure 3, which shows that the former solution (i.e., the $x_{1}=0.128,x_{2}=x_{3}=0.268$ roots) provides the steepest plot.

### 2.2. The Compressed Chebyshev Polynomial Method (CoCPM)

The key concept of the novel method is that the Chebyshev polynomial (or any other polynomial) is compressed in the amplitude and frequency range to: (a) satisfy the criterion which requires that its maximum local peaks (in absolute value) do not exceed 1 and (b) cross the (1,1) point. In particular, via the corresponding plot, we find which combination of the Chebyshev polynomial root variations leads to its maximum absolute value, i.e., the maximum of all the maximum local peaks. Then, we compress the polynomial both in amplitude (so that the above maximum absolute value becomes 1) and in frequency (so that the polynomial crosses the (1,1) point). We designate the final outcome as the compressed Chebyshev polynomial (CoCP) and describe its derivation in detail below.

Let us suppose a *n*th-order Chebyshev polynomial, $C_{n}\left(x\right)$, expressed, in the case of even symmetry (with a similar analysis for odd symmetry), as $C_{n}\left(x\right)=\prod_{i=1}^{n/2}\left(x^{2}-x_{i}^{2}\right)$, whose known roots are $0\leq x_{1}\leq \cdots \leq x_{n/2}\leq 1$. Next, we introduce factor *k* and coefficient a<1, such that the maximum deviation of $|kC_{n}\left(x/a\right)|$ from the horizontal axis (due to the variation of its roots) does not exceed 1 and $C_{n}\left(x/a\right)$ crosses the (1,1) point. This, in turn, means that $kC_{n}\left(1/a\right)=1$ and $k=1/C_{n}\left(1/a\right)$, respectively. Actually, *a* is used to retain the same bandpass zone. If the maximum deviation of $kC_{n}\left(x/a\right)$ from the horizontal axis occurs at the *i*th peak, we define the following distorted polynomial

(20) $$\begin{array}{c} kC_{n}^{\left(i\right)}\left(x\right)=k\prod_{k=1}^{i-1}\left\{{\left[\left(x/a\right)+d_{k}\right]}^{2}-x_{k}^{2}\right\}\prod_{k=i}^{n/2}\left[{\left(x/a\right)}^{2}-{\left(x_{k}+d_{k}\right)}^{2}\right], \end{array}$$

so that its maximum value, i.e., 1, is at its *i*th peak, for $x=\xi_{i}$. Moreover, the unknown *a* and $\xi_{i}$ are determined by the system of

(21) $$\begin{array}{cc} k\left|\max_{i}C_{n}^{\left(i\right)}\left(x\right)\right| & ={\left(-1\right)}^{(n/2)+1-i}\prod_{k=1}^{i-1}\left[{\left(\xi_{i}+d_{k}\right)}^{2}-{\left(ax_{k}\right)}^{2}\right]\\ \; & \times \prod_{k=i}^{n/2}\left[\xi_{i}^{2}-{\left(ax_{k}+d_{k}\right)}^{2}\right]/\prod_{i=1}^{n/2}\left[1-{\left(ax_{i}\right)}^{2}\right]=1 \end{array}$$

and

(22) $$\begin{array}{cc} {\left.\frac{\partial C_{n}^{\left(i\right)}\left(x\right)}{\partial x}\right|}_{x=\xi_{i}} & \equiv \sum_{j=1}^{i-1}\left\{\left(\xi_{i}+d_{j}\right)\prod_{k=1,\neq j}^{i-1}\left[{\left(\xi_{i}+d_{k}\right)}^{2}-{\left(ax_{k}\right)}^{2}\right]\prod_{k=i}^{n/2}\left[\xi_{i}^{2}-{\left(ax_{k}+d_{k}\right)}^{2}\right]\right\}\\ \; & +\xi_{i}\sum_{j=i}^{n/2}\left\{\prod_{k=1}^{i-1}\left[{\left(\xi_{i}+d_{k}\right)}^{2}-{\left(ax_{k}\right)}^{2}\right]\prod_{k=i,\neq j}^{n/2}\left[\xi_{i}^{2}-{\left(ax_{k}+d_{k}\right)}^{2}\right]\right\}=0. \end{array}$$

In this framework, the desired CoCP is denoted as

(23) $$\begin{array}{c} H_{n}\left(x\right)=\prod_{i=1}^{n/2}\left[x^{2}-{\left(ax_{i}\right)}^{2}\right]/\prod_{i=1}^{n/2}\left[1-{\left(ax_{i}\right)}^{2}\right]. \end{array}$$

Although $H_{n}\left(x\right)$ is defined in [−1,1], it remains a typical Chebyshev polynomial in [−a,a], where its roots are the $ax_{i}$ and its maximum value is

(24) $$\begin{array}{c} H_{n}\left(0\right)=\prod_{i=1}^{n/2}{\left(ax_{i}\right)}^{2}/\prod_{i=1}^{n/2}\left[1-{\left(ax_{i}\right)}^{2}\right]. \end{array}$$

Notice that, in (a,1], Equation (23) increases until it crosses the (1,1) point.

The solution of the proposed polynomial is always very close to the optimal one, accomplishing, also, a much simpler filtering process and filter implementation, which can be readily utilized for the improvement of existing techniques, as shown in Section 3. Due to the theorem of [43], the roots of Equation (23) are calculated from the distorted $H_{n}^{\left(i\right)}\left(x\right)$ polynomials via $|\max_{i}H_{n}^{\left(i\right)}\left(x\right)|=p_{i}$, with $p_{i}<1$ (at least one of them), for 1≤i≤n/2. Hence, the optimal solution is obtained from $p_{i}\leq 1$, which yields a definitely steeper plot.

**Figure 4:**
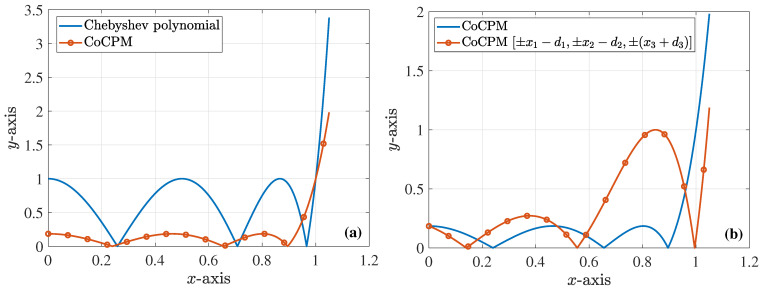
Diverse sixth-order polynomial realizations for the design of the corresponding filter (absolute values are depicted). (**a**) The Chebyshev polynomial and the polynomial derived via the proposed CoCPM, with $d_{1}=d_{2}=d_{3}=0.1$. (**b**) Two different polynomials, derived via the proposed CoCPM, with $d_{1}=d_{2}=d_{3}=0.1$ and the $\pm x_{1}-d_{1},\pm x_{2}-d_{2},\pm \left(x_{3}+d_{3}\right)$ combination of the roots, respectively. The third maximum peak does not exceed 1.

**Figure 5:**
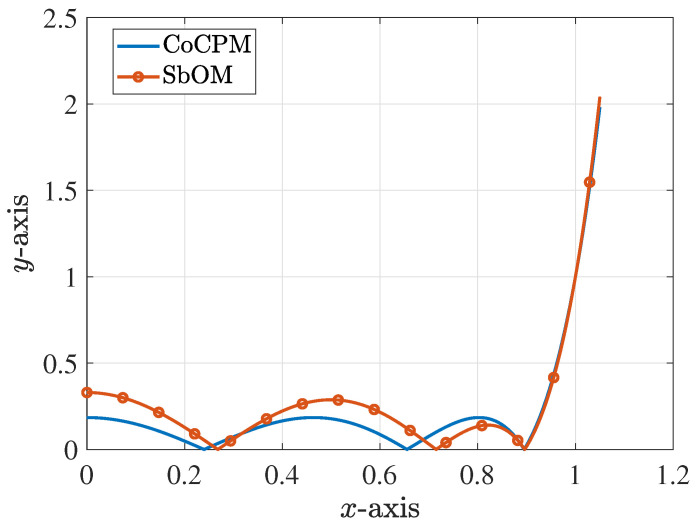
Two sixth-order polynomial realizations (absolute values are depicted), derived via the proposed SbOM and CoCPM, with $d_{1}=d_{2}=d_{3}=0.1$.

As an example, lets us consider the case of a sixth-order filter, with $d_{1}=d_{2}=d_{3}=0.1$. The resulting Chebyshev polynomial obtains its maximum value for the $x_{1}-d_{1},x_{2}-d_{2},x_{3}+d_{3}$ root variation, i.e., at the third peak. Then, by employing Equations (21) and (22), we obtain $\xi_{3}=0.8485$ and a=0.9266, so that the roots of the CoCP are $ax_{1}=0.2398,ax_{2}=0.6552,ax_{3}=0.895$, where $x_{i}$ are the roots of the Chebyshev polynomial. It is must be stated that the optimal solution is acquired with all $p_{i}=1$, which is better than the CoCPM, where only $p_{3}=1$. Lastly, and through Equation (24), the maximum value of the new polynomial, in [−0.9266,0.9266], is found to be 0.1847. The prior outcomes are shown in Figure 4, while Figure 5 compares the derived polynomials through the SbOM and CoCPM.

### 2.3. Extension to Rational Filtering Functions

A noteworthy asset of the SbOM is its straightforward application to rational polynomial functions. For this aim, let us presume the prototype rational polynomial function $F_{n}\left(x\right)$, with zeros (real roots) $x_{i}\in \left[-1,1\right]$ and poles $y_{i}\notin \left[-1,1\right]$. Moreover, we assume that the maximum value of $F_{n}\left(x\right)$ in [−1,1] does not exceed 1, it crosses the (1,1) point, and its fluctuations outside [−1,1] are not below 100. The threshold of 100 is justified by considering that for ε=0.1 (so that in the passband $|S_{11}|\leq -20\;$dB), we should have $|S_{12}|\leq -20\;$dB in the out-of-band zone, which results in $|F_{n}\left(x\right)|\geq 100$, for |x|>1. Then, $F_{n}\left(x\right)$ is derived by presuming that any variations of its zeros and poles do not enforce $|F_{n}\left(x\right)|>1$ in [−1,1] and $|F_{n}\left(x\right)|<100$ outside [−1,1]. Hence, the *i*th maximum peak in [−1,1] occurs for the $x_{1}-d_{1},\ldots ,x_{i}-d_{i},x_{i+1}+d_{i+1},\ldots ,x_{n}+d_{n}$ combinations of the zeros and the $y_{1}+d_{1},\ldots ,y_{n/2}+d_{n/2},y_{(n/2)+1}-d_{(n/2)+1},\ldots ,y_{n}-d_{n}$ combinations of the poles, i.e., the poles approach the *i*th peak. Similarly, the lowest *i*th valley outside [−1,1] occurs for the $x_{1}+d_{1},\ldots ,x_{n}+d_{n}$ combinations of the zeros and the $y_{1}-d_{1},\ldots ,y_{n/2}-d_{n/2},\ldots ,y_{i-1}-d_{i-1},y_{i+1}+d_{i+1},\ldots ,y_{n}+d_{n}$ combinations of the poles, i.e., the zeros approach the *i*th valley. In fact, due to the close proximity of zeros to poles and the steep transit zone, the aforementioned scheme is efficient for small changes, namely the rational function is more sensitive to zeros and poles variations than the polynomial. However, the principal notion for deriving the $F_{n}\left(x\right)$, such that its peaks and valleys are not over predetermined values, is still valid. Observe that the optimization theorem of [43] is applicable to the $|\partial F_{n}{\left(x\right)/\partial x|}_{x=1}$ derivative, owing to the steep plot of the transit zone.

**Figure 6:**
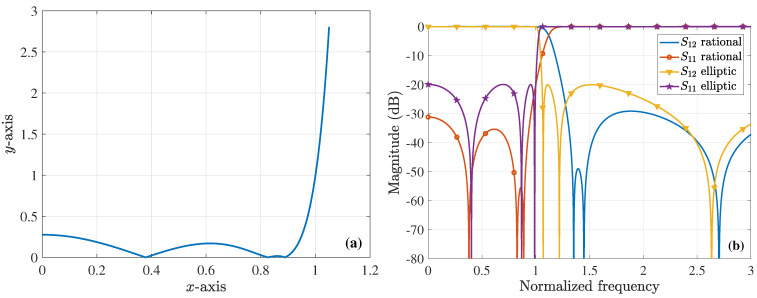
Design of a sixth-order rational filter. (**a**) The sixth-order (sixth-order polynomial for the numerator and the denominator) prototype rational filtering function (absolute values depicted) derived by considering that the zeros and poles vary by ±0.1 (all $d_{i}$s are equal to 0.1). (**b**) Magnitude of the *S*-parameters (ε=0.1), extracted through the sixth-order elliptic polynomial and the rational filtering function of Figure 6a.

**Figure 7:**
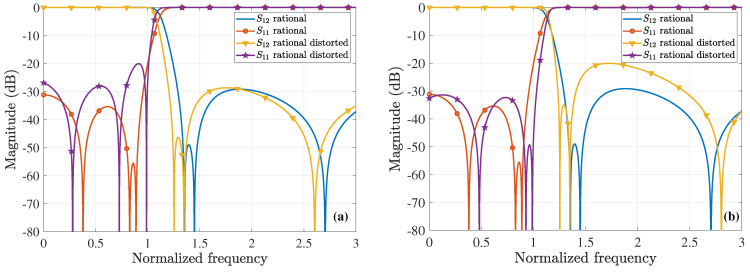
Magnitude of the *S*-parameters (ε=0.1) for a sixth-order rational filter, extracted through the rational filtering function of Figure 6a and when the filter is distorted by the combination of (**a**) $\pm x_{1}-d_{1},\pm \left(x_{2}+d_{2}\right),\pm \left(x_{3}+d_{3}\right)$ zeros and $\pm \left(y_{1}-d_{1}\right),\pm \left(y_{2}-d_{2}\right),\pm \left(y_{3}-d_{3}\right)$ poles ($|S_{11}|$ never exceeds the level of −20dB) and (**b**) $\pm x_{1}+d_{1},\pm x_{2}+d_{2},\pm x_{3}+d_{3}$ zeros and $\pm y_{1}-d_{1},\pm \left(y_{2}+d_{2}\right),\pm \left(y_{3}+d_{3}\right)$ poles ($|S_{12}|$ never exceeds the level of −20dB in the out-of-band zone).

Next, we design a sixth-order elliptic filter, whose polynomial function has the the $x_{1}=0.4017,x_{2}=0.868,x_{3}=0.9898$ zeros and the $y_{1}=1.0691,y_{2}=1.2192,y_{3}=2.6344$ poles ([44] see pp. 33, 34 and replace *m* with $m^{1/2}$ in (2.5.19) and (2.5.20)). Assume, for instance, that all $d_{i}$s are equal to 0.1, i.e., the roots and poles change by ±0.1. This leads to a sixth-order prototype rational filtering function with $x_{1}=0.379,x_{2}=0.827,x_{3}=0.888$ zeros and $y_{1}=1.353,y_{2}=1.448,y_{3}=2.704$ poles, as shown in Figure 5a. Its solution is obtained by solving Equations (8)–(10) sequentially for $x_{1},\xi_{2},x_{2},\xi_{3},x_{3},\xi_{4},y_{1},\xi_{5},y_{2}$. Then, $y_{3}$ is calculated by imposing the $K=\left(1-y_{1}^{2}\right)\left(1-y_{2}^{2}\right)\left(1-y_{3}^{2}\right)/\left[\left(1-x_{1}^{2}\right)\left(1-x_{2}^{2}\right)\left(1-x_{3}^{2}\right)\right]=100$ coefficient via the $\lim_{x\to \infty }F_{n}\left(x\right)=K$ constraint. Moreover, $\xi_{4}$ and $\xi_{5}$ are the abscissas of the lowest valleys between $y_{1},y_{2}$ and $y_{2},y_{3}$ poles, respectively. These interesting findings are presented in Figure 6 and Figure 7, which, also, include the case when the prior filter is distorted by certain combinations of the roots. As detected, the featured extension to rational polynomial functions provides a promising treatment for several complicated distortions, thus guaranteeing a reliable realization process for effective and robust filters.

## 3. Realistic Applications and Numerical Verification

For its comprehensive validation, the novel methodology is applied to the design of several waveguide and microstrip line filters, which constitute indispensable elements of contemporary 6G communication systems. The selected filters operate at fairly different frequencies, covering the popular 6G spectrum between 2GHz and 65GHz, in order to prove that our technique does not depend on frequency. In this context, the required polynomials are derived through the proposed SbOM and CoCPM of Section 2, while all numerical simulations are performed via the COMSOL Multiphysics^®^ simulation software [38].

### 3.1. Design of a Sixth-Order Waveguide Filter

To derive the sixth-order polynomial for the specific waveguide filter, we, firstly, estimate the variations of the roots from the relevant literature. Then, we compare our SbOM and CoCPM solutions with the chained function filter scheme [45] and the filter presented in [37]. It is should be emphasized that this comparison is conducted according to a unified way, proposed herein, in order to draw trustworthy conclusions, namely:

- The bandpass zone is defined in [−1,1] and all polynomials must cross the (1,1) point.
- The maximum amplitude of each prototype polynomial, as distorted owing to the variation of its roots, should be 1.

The required estimation on the variation of the roots is accomplished via the plots in [45], where a distorted sixth-order Chebyshev polynomial is depicted. Note that the position of the roots refers to the abscissas of the minima of the $S_{11}$-parameter magnitude, for which we derive that $d_{1}\in \left[-0.2588,0.02\right],d_{2}\in \left[-0.07,0.07\right]$, and $d_{3}\in \left[-0.14,0.1\right]$. Moreover, to consider the worst case scenario, we assume that $d_{1},d_{2},d_{3}$ can move independently to each other, and hence the diverse elevations of the peaks may be larger than those in [45].

Subsequently, by means of the SbOM and Equations (8)–(10), the roots of the optimal polynomial $F_{6}\left(\omega \right)$ are calculated as $x_{1}=0.3993,x_{2}=0.6875,x_{3}=0.8832$. Moreover, through the CoCPM and Equations (21) and (22), the respective roots are $x_{1}=0.2363,x_{2}=0.6456,x_{3}=0.8819$. For the latter solution, the maximum amplitude occurs for the third peak that should be equal to 1, i.e., we solve Equations (21) and (22) for the $x_{1}-0.2588,x_{2}-0.07,x_{3}+0.1$ root variation. Indeed, through the proper plots, it can be promptly detected that all the other peaks have lower maximum values due to the fluctuations of the roots. Furthermore, the maximum of the prior CoCP in [−a,a], with a=0.913, is found to be 0.1479 from Equation (24). So, the waveguide filter is equivalent to a Chebyshev filter with $\varepsilon^{\prime }=0.1479\varepsilon =0.01486$ in [−a,a]. This means that we lower the plot of the $S_{11}$-parameter magnitude by an additional 16.5dB (from the initial −20dB), whereas the (1,1) point continues to correspond to the level of −20dB.

**Figure 8:**
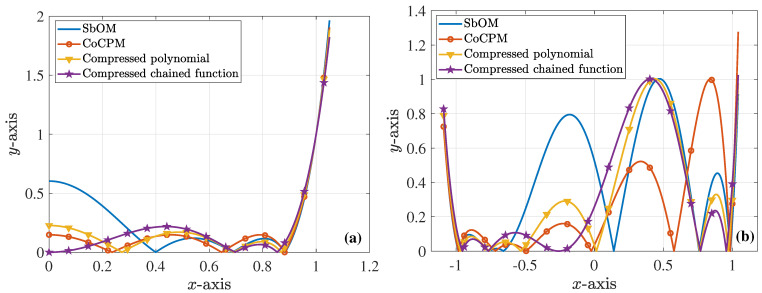
Optimal sixth-order polynomial, $F_{6}\left(\omega \right)$, realizations for the design of the corresponding waveguide filter, derived via the proposed SbOM and CoCPM, the compressed polynomial [37], and the compressed chained function [45] (absolute values are depicted). (**a**) The initial form of the different $F_{6}\left(\omega \right)$. All plots are almost identical for |x|>1. (**b**) Behavior of the diverse $F_{6}\left(\omega \right)$ owing to the variation of their roots. The maximum amplitudes do not exceed 1.

**Figure 9:**
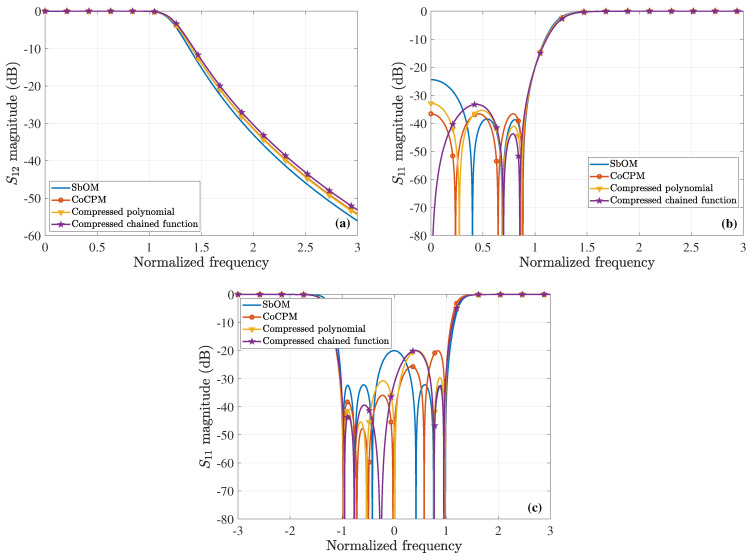
Magnitude of the *S*-parameters (ε=0.1) for the 6th-order waveguide filter. (**a**) $|S_{12}|$ and (**b**) $|S_{11}|$, both extracted through the corresponding polynomials of Figure 8a. The results of the CoCPM and the compressed polynomial [37] coincide for |x|>1. (**c**) $|S_{11}|$ owing to the variation of the corresponding $F_{6}\left(\omega \right)$ roots of Figure 8b ($|S_{11}|$ never exceeds the level of −20dB).

For our comparisons, we employ the chained function of [45], which combines the first-, second-, and third-order Chebyshev polynomials. Concerning the specific sixth-order waveguide filter, this results in $8x^{6}-10x^{4}+3x^{2}=8x^{2}\left(x^{2}-0.70712\right)\left(x^{2}-0.8662\right)$ that crosses the (1,1) point. Since the maximum value of its second peak is larger than 1, the chained function is compressed in frequency and amplitude, by means of Equations (21) and (22), thus leading to the 0,0.6972, and 0.8539 roots. Next, we consider the polynomial presented in [37], whose roots are 0.3036,0.7683,0.9659 and its largest peak, due to root variation, is the second one. However, the specific function does not cross the (1,1) point; therefore, we compress it in frequency and amplitude, via Equations (21) and (22), to acquire the 0.2731,0.6911, and 0.8688 roots. Summarizing all the optimal $F_{6}\left(\omega \right)$, Figure 8a illustrates their initial plots, which practically coincide for |x|>1. Conversely, Figure 8b shows their behavior owing to the variation of their roots, as discussed in the previous paragraph. Note that none of the $F_{6}\left(\omega \right)$ maximum amplitudes surpass 1. In addition, Figure 9a,b present the magnitude of the $S_{12}$-parameter (ε=0.1) and the $S_{11}$-parameter (ε=0.1), respectively, both computed in terms of the corresponding polynomials of Figure 8a. It can be detected that the outcomes of the proposed CoCPM and the compressed polynomial [37] are practically identical for |x|>1. Lastly, the impact of the various $F_{6}\left(\omega \right)$ root variations of Figure 8b on the $|S_{11}|$ (ε=0.1) is examined in Figure 9c. Therefore, we prove that none of the plots exceeds the level of −20dB, unlike typical formulations that lack to offer adequate results. In this manner, one can comprehend the merits of our method which, together with the compression concept for the significant improvement of existing approaches, can provide flexible 6G filters.

### 3.2. Design of a Fourth-Order Chebyshev Microstrip Line Filter at 2.4GHz

The verification of our theoretical framework, herein, focuses on the analysis of a microstrip line filter from the Chebyshev family, which comprises a frequent selection in modern communication systems. Therefore, after studying the filter in its initial form, we deform the microstrip line to emulate potential fabrication tolerances and then compute the new filtering function, via our methodology, which leads to the new microstrip filter. Finally, the resulting structure is deformed, again, in order to systematically investigate its overall behavior and substantiate the effectiveness of the featured design procedure.

**Figure 10:**
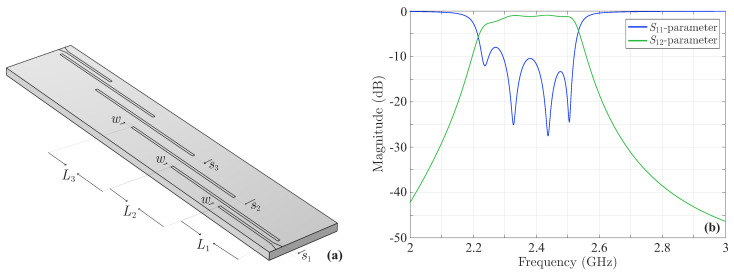
The initial form of the fourth-order Chebyshev microstrip line filter terminated by means of a taper at both sides for the necessary input–output signal routing. (**a**) Geometry and (**b**) magnitude of the *S*-parameters. The maximum value of the $|S_{11}|$ first peak is −7.98dB.

Based on these aspects, we study the fourth-order Chebyshev microstrip line filter of Figure 10a with a central frequency of 2.4GHz, described in [46]. In its initial form, the dimensions of the filter are $L_{1}=11.4\;$mm, $L_{2}=L_{3}=11.5\;$mm, w=0.4mm, $s_{1}=0.7\;$mm, $s_{2}=1.6\;$mm, and $s_{3}=1.8\;$mm. Also, tapers are used at the input–output ends to reduce the fringing fields, due to the transition from the 50Ω input microstrip lines to the thinner coupled lines of the structure. Our basic design restriction is $|S_{11}|<-9.636\;$dB. As previously described, this implies that the filtering function is given by $8\left(x^{2}-0.383^{2}\right)\left(x^{2}-0.924^{2}\right)$, with ε=0.35 in the $|S_{12}|$ formula. Therefore, and after the appropriate numerical simulations, Figure 10b presents the magnitude of the *S*-parameters, which, as promptly observed, are not symmetrical, with the first $|S_{11}|$ peak computed at −7.98dB.

**Figure 11:**
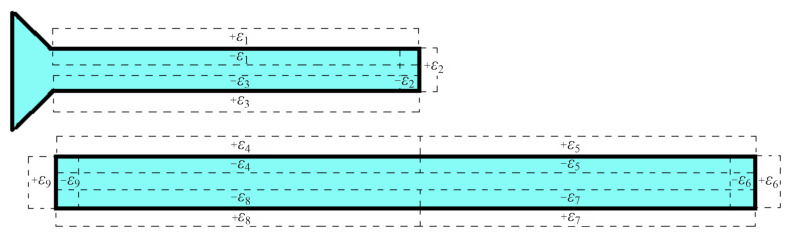
Geometry of the deformed, due to fabrication imperfections, coupled microstrip lines of the fourth-order Chebyshev filter of Figure 10a. The sketch illustrates the top right-hand part of the filter and the dashed lines depict the deformations at the microstrip lines.

**Figure 12:**
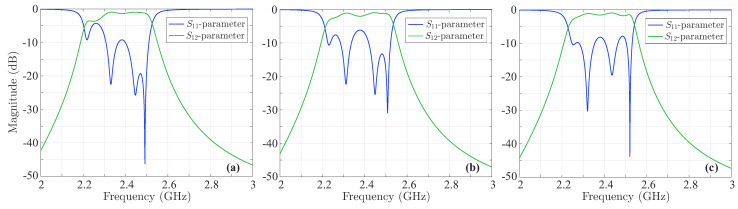
Magnitude of the *S*-parameters of the deformed fourth-order Chebyshev microstrip line filter. The deformation occurs according to a sequence of $\varepsilon_{i}$s, such that the $|S_{11}|$ first, second, or third peak is maximized. Explicitly, the maximum value of the $|S_{11}|$ (**a**) first peak is −4.27dB, (**b**) second peak is −6.16dB, and (**c**) third peak is −7.88dB.

Subsequently, the prior filter is deformed both in terms of the width and length of its microstrip lines. For example, let us consider Figure 11, which presents the top right-hand part of the filter shown in Figure 10a. In particular, width deformations are created by adding $\pm \varepsilon_{i}=30\;\mu$m (for i=1,3,4,5,7,8), while length deformations by adding $\pm \varepsilon_{i}=30\;\mu$m (for i=2,6,9). Moreover, regarding the inner microstrip lines, there exist six possible ways of deformation, i.e., six $\varepsilon_{i}$s: one for each of their short edges and two for each of their long ones. On the other hand, concerning the outer microstrip lines, there are three possible ways of deformation, i.e., three $\varepsilon_{i}$s: one for each of their short edges and one for each of their long ones. Hence, the total number of deformations is 30. Furthermore, to avoid excessive simulations and motivated by the sensitivity function for parasitic elements of [47], we presume a Taylor series dependence of any filter characteristic, ch, on the $\varepsilon_{i}$ deformation, as

(25) $$\begin{array}{c} ch=\sum_{i=1}^{30}\alpha_{i}\varepsilon_{i}+\sum_{i=1}^{30}\gamma_{i}\varepsilon_{i}^{2}+\mathrm{terms}\mathrm{of}\mathrm{second}\mathrm{degree}\mathrm{or}\mathrm{greater}, \end{array}$$

where $\alpha_{i}$ abd $\gamma_{i}$ are unknown weights. Note that, throughout our analysis, we have selected ch to stand for the local maximum of the $S_{11}$-parameter magnitude, although equivalent outcomes could be derived if, for example, ch represented a root of the filtering function. Considering these notions, we perform two numerical simulations for every $\varepsilon_{i}$ (i.e., for the $+\varepsilon_{i}$ and $-\varepsilon_{i}$ value), setting the rest of the $\varepsilon_{i}$s to zero, and record each local maximum. Actually, we keep the $\varepsilon_{i}$ for which the local peak is maximized, namely the $+\varepsilon_{i}$, $-\varepsilon_{i}$, or $\varepsilon_{i}=0$ case. Thus, a total of 60 simulations are conducted and three sequences of $\varepsilon_{i}$s are obtained to maximize each local peak. Finally, we simulate the deformed filter for these sequences and derive the local peaks of the $|S_{11}|$ to be maximized, as illustrated in Figure 12. In particular, the maximum value of the $|S_{11}|$ first peak (Figure 12a) is −4.27dB, second peak (Figure 12b) is −6.16dB, and third peak (Figure 12c) is −7.88dB, none of which, however, fulfils our initial $|S_{11}|<-9.636\;$dB design requirement. These maxima occur for the (−0.171,0.025,0.074,−0.104), (−0.054,−0.138,0.09,0.024), and (0.152,−0.06,−0.008,0.128) sequences of root deviations, respectively (the numbers in parentheses denote the deviation of the first, second, third, and fourth root of the initial filter, normalized to [−1,1]).

**Figure 13:**
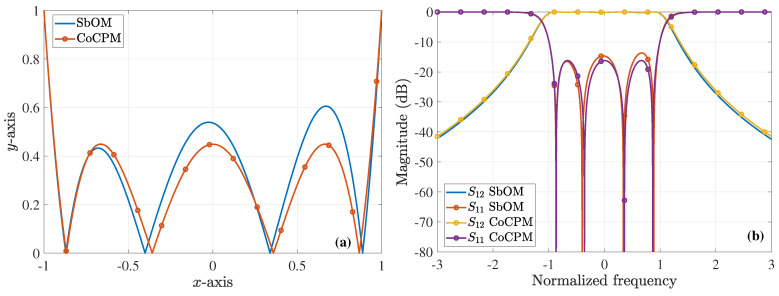
(**a**) The new fourth-order filtering function derived via the SbOM and the CoCPM and (**b**) magnitude of the respective *S*-parameters.

**Figure 14:**
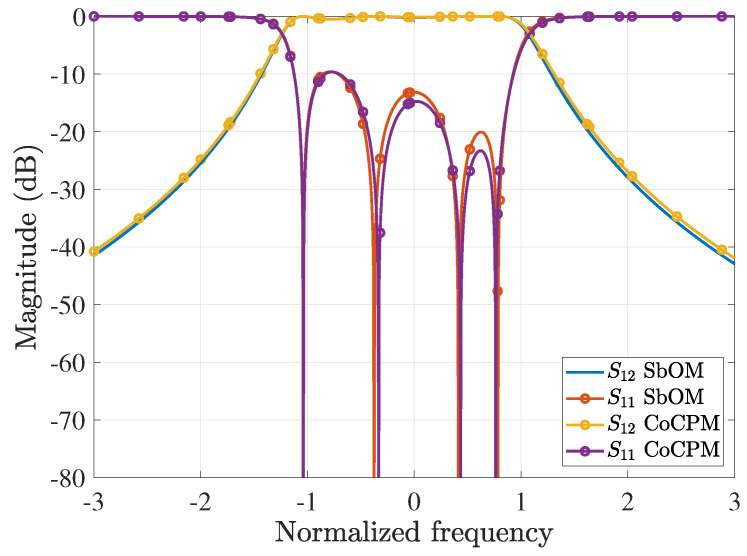
Magnitude of the *S*-parameters of the new filtering functions in Figure 13a, distorted for the first maximum local peak of the $|S_{11}|$.

For these sequences of root deviations, we, next, calculate the new filtering function, on condition that none of the maximum $|S_{11}|$ local peaks exceeds the already prefixed level of −9.636dB. This fourth-order filtering function, retrieved via the SbOM, is 5.11(x+0.87)(x+0.401)(x−0.339)(x−0.887), while the one, extracted through the CoCPM, is $4.629\left(x^{2}-0.359^{2}\right)\left(x^{2}-0.867^{2}\right)$, as depicted in Figure 13a. For both methods, we set ε=0.35 in the $|S_{12}|$ formula. In addition, Figure 13b shows the magnitude of the *S*-parameters, computed by means of the prior filtering functions, from which it can be readily deduced that the $|S_{11}|<-9.636\;$dB prerequisite is fully satisfied. To certify the efficiency of our methodology, let us, for instance, concentrate on the maximization of the first $|S_{11}|$ local peak. Since this maximum is attributed to the (−0.171,0.025,0.074,−0.104) sequence, the distorted filtering function (in the prototype) is: (a) 5.11(x+0.87+0.171)(x+0.401−0.025)(x−0.339−0.074)(x−0.887+0.104) for the SbOM and (b) 4.629(x+0.867+0.171)(x+0.359−0.025)(x−0.359−0.074)(x−0.867+0.104) for the CoCPM. The magnitude of the corresponding *S*-parameters is presented is Figure 14, which substantiates that the first maximum $|S_{11}|$ local peaks of both distorted polynomials do not exceed the −9.636dB level of the initial filter.

**Figure 15:**
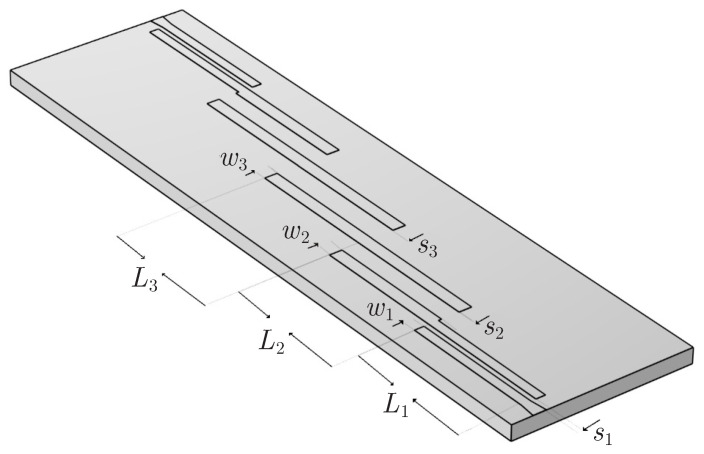
Geometry of the new fourth-order Chebyshev microstrip line filter designed via the CoCPM-extracted filtering function of Figure 13a.

**Figure 16:**
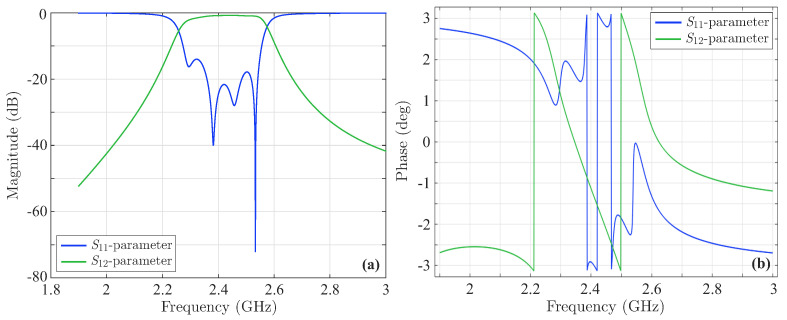
The *S*-parametrs of the new fourth-order Chebyshev microstrip line filter of Figure 15. (**a**) Magnitude and (**b**) phase. The bandwidth of the filter is 9.387% and its ripple 0.105dB.

**Figure 17:**
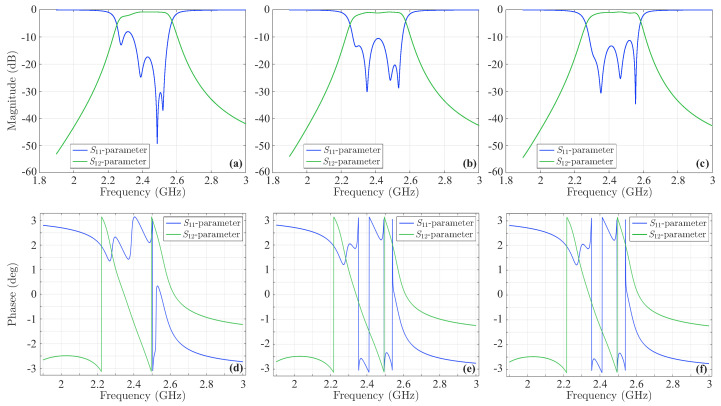
Magnitude and phase of the *S*-parameters of the new deformed fourth-order Chebyshev microstrip line filter. The maximum value of the $|S_{11}|$ (and the corresponding phase) (**a**,**d**) first peak is −8.05dB, (**b**,**e**) second peak is −10.4dB, and (**c**,**f**) third peak is −10.92dB. All peaks are below the levels of their initial filter counterparts.

The last stage of the validation process involves the design of the new fourth-order Chebyshev microstrip line filter of Figure 15, consistent with the filtering function derived above via the CoCPM and shown in Figure 13a. Basically, this filtering function leads to a filter with a bandwidth of 9.387% and a ripple of 0.105dB. To calculate the symmetric odd-mode, $Z_{oi}$, and even-mode, $Z_{ei}$, impedances (for i=1,2,3) of the device, we employ the well-known formulae from ([12] i.e., (8.108) and (8.121), respectively). Thus, we obtain $Z_{o1}=38.4426\;\Omega$, $Z_{e1}=74.7126\;\Omega$, $Z_{o2}=44.6487\;\Omega$, $Z_{e2}=56.8367\;\Omega$, and $Z_{o3}=45.6354\;\Omega$, $Z_{e3}=55.2984\;\Omega$. Moreover, the dimensions of the structure in Figure 15 are calculated by means of the relevant well-known expressions, found in [12,48]. So, after some mathematical manipulations, one acquires $L_{1}=11.3593\;$mm, $L_{2}=11.0938\;$mm, $L_{3}=11.0615\;$mm, $w_{1}=0.801\;$mm, $s_{1}=0.415\;$mm, $w_{2}=1.074\;$, $s_{2}=1.494\;$mm, $w_{3}=1.089\;$mm, and $s_{3}=1.819\;$mm. The promising performance of the numerically simulated filter is verified through the magnitude of its *S*-parameters in Figure 16a, which indicate the significantly reduced $|S_{11}|$ local peaks and the considerable bandwidth. Similar deductions can be drawn from the correspondingly enhanced phase of the filter’s *S*-parameters in Figure 16b. Finally, the new filter is deformed, again, via the same sequence of microstrip line deformation, already applied to the initial structure. Figure 17 presents the magnitude and the phase of the *S*-parameters. Hence, the maximum value (and the corresponding phase) of the $|S_{11}|$ first peak (Figure 17a,d) is −8.05dB, second peak (Figure 17b,e) is −10.4dB, and third peak (Figure 17c,f) is −10.92dB. As observed, apart from the slight elevation of the first peak, attributed, chiefly, to design inaccuracies, the rest of the peaks do not exceed the limitation of −9.696dB. Furthermore, all the peaks are definitely below the levels of their initial filter counterparts. These observations verify the robustness of the featured filter (even if deformed) and the efficiency of our technique to treat construction imperfections in demanding design scenarios.

### 3.3. Design of a Fourth-Order Microstrip Parallel Coupled-Line Bandpass Filter at 62.5 GHz

Having successfully determined the efficiency of our technique at the low part of the 6G spectrum, we extend our investigation to much higher frequencies. In this manner, we will be able to prove that its overall performance is completely independent of the filter’s operating frequency and certify its reliable use at any frequency range. As already described in the previous examples, after studying the filter in its initial form, the microstrip line is deformed to imitate potential fabrication tolerances. Next, we compute the new filtering function, which leads to the new (and far more resilient to fabrication imperfections) microstrip filter. The resulting device is then deformed, again, in order to thoroughly assess its overall behavior and affirm the advantages of the design process.

**Figure 18:**
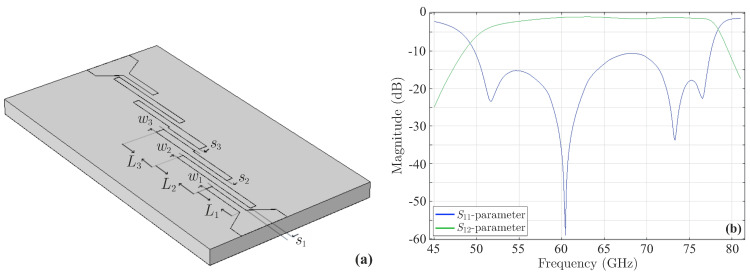
The initial form of the fourth-order microstrip parallel coupled-line bandpass filter terminated by means of a taper at both sides for the appropriate input–output signal routing. (**a**) Geometry and (**b**) magnitude of the *S*-parameters. The maximum value of the $|S_{11}|$ first peak is −10.59dB.

Hence, let us examine the fourth-order microstrip parallel coupled-line bandpass filter, depicted in its initial form in Figure 18a, with a central frequency at 62.5GHz, as presented in [49]. Actually, such devices, operating in the vicinity of 60GHz, have triggered a significant research interest, due to anticipated impressive usage of these frequencies in a multitude of future scenarios [1,2,19]. The dimensions of the filter are $L_{1}=0.995934\;$mm, $L_{2}=0.924559\;$mm, $L_{3}=0.984250\;$mm, $w_{1}=0.643128\;$mm, $w_{2}=0.604012\;$mm, $w_{3}=0.642112\;$mm, $s_{1}=0.079248\;$mm, $s_{2}=0.062992\;$mm, and $s_{3}=0.078740\;$mm. Its numerical simulation leads to the outcomes of Figure 18b, which illustrates the magnitude of the *S*-parameters. Observe that all $|S_{11}|$ peaks are below the desired threshold of −10dB.

**Figure 19:**
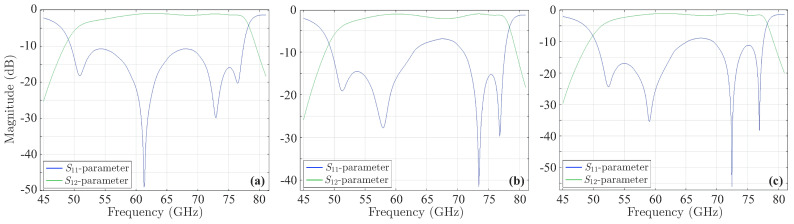
Magnitude of the *S*-parameters of the deformed microstrip parallel coupled-line bandpass filter. The deformation occurs according to a sequence of $\varepsilon_{i}$s, such that the $|S_{11}|$ first, second, or third peak is maximized. Specifically, the maximum value of the $|S_{11}|$ (**a**) first peak is −10.65dB, (**b**) second peak is −6.84dB, and (**c**) third peak is −11.02dB.

To apply our method, the prior filter is, next, deformed by adding $\pm \varepsilon_{i}=5\;\mu$m (for i=1,2,…,30) to each of its microstrip lines. As a consequence, a total of 60 simulations are performed and three sequences of $\varepsilon_{i}$’s are acquired in order to maximize each local peak. Then, the deformed filter is computationally simulated for these sequences to extract the local peaks of the $|S_{11}|$ to be maximized, which are shown in Figure 19. Explicitly, the maximum value of the $|S_{11}|$ first peak (Figure 19a) is −10.65dB, second peak (Figure 19b) is −6.84dB, and third peak (Figure 19c) is −11.02dB.

**Figure 20:**
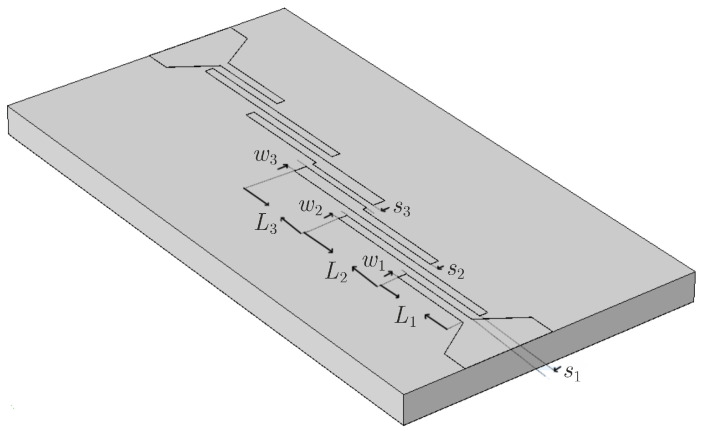
Geometry of the new fourth-order microstrip parallel coupled-line bandpass filter designed via the proposed methodology.

**Figure 21:**
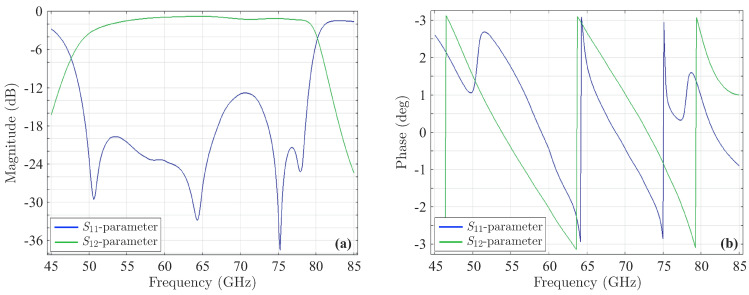
The *S*-parametrs of the new fourth-order microstrip parallel coupled-line bandpass filter. (**a**) Magnitude and (**b**) phase. The bandwidth of the filter is 41.331% and its ripple 0.1651dB.

**Figure 22:**
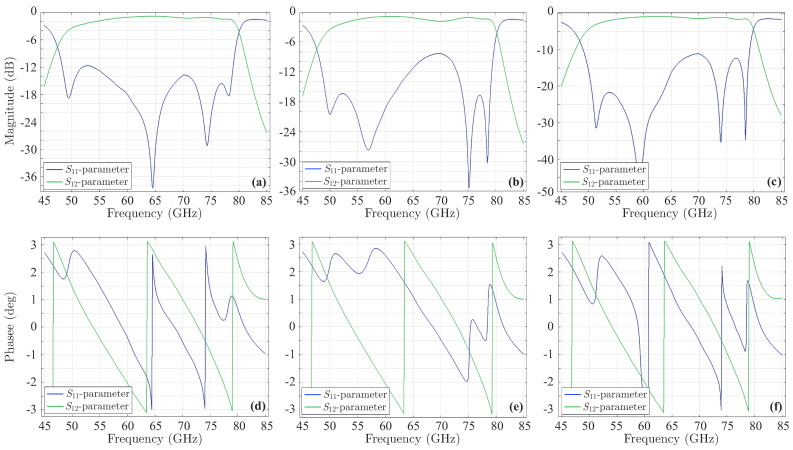
Magnitude and phase of the *S*-parameters of the new deformed fourth-order microstrip parallel coupled-line bandpass filter. The maximum value of the $|S_{11}|$ (and the corresponding phase) (**a**,**d**) first peak is −11.6dB, (**b**,**e**) second peak is −8.2dB, and (**c**,**f**) third peak is −12.27dB.

The preceding results lead to the design of the new fourth-order microstrip parallel coupled-line bandpass filter, provided in Figure 20. The filter has a bandwidth of 41.331% and a ripple of 0.1651dB. By computing the symmetric odd-mode and even-mode impedances, as in the our second example, one obtains $Z_{o1}=39.9755\;\Omega$, $Z_{e1}=112.2265\;\Omega$, $Z_{o2}=37.5069\;\Omega$, $Z_{e2}=88.6819\;\Omega$, and $Z_{o3}=37.8822\;\Omega$, $Z_{e3}=79.1382\;\Omega$. If we recall the expressions of [12] and [48], the dimensions of the new filter are $L_{1}=0.758\;$mm, $L_{2}=0.907\;$mm, $L_{3}=0.753\;$mm, $w_{1}=0.075\;$mm, $w_{2}=0,075\;$mm, $w_{3}=0.110\;$mm, $s_{1}=0,072\;$mm, $s_{2}=0.072\;$mm, and $s_{3}=0.06\;$mm. For this design, the numerically derived magnitude and phase of its *S*-parameters are presented in Figure 21. Note that all $|S_{11}|$ exhibit a significant reduction of their local peaks, compared with those of the initial filter. In order to complete our analysis, we employ the same sequence of microstrip line uncertainties, as in the initial filter of Figure 18a, to deform the new filter and display the magnitude and phase of its *S*-parameters in Figure 22. Thus, the maximum value (and the respective phase) of the $|S_{11}|$ first peak (Figure 22a,d) is −11.6dB, second peak (Figure 22b,e) is −8.2dB, and third peak (Figure 22c,f) is −12.27dB. Note that all the peaks of the new filter are below the respective values of the initial structure (i.e., enhanced durability to construction imperfections), despite the notably high central frequency. This proves that the performance of the proposed algorithm is not only very satisfactory but, also, completely independent of the filter’s operating frequency.

## 4. Conclusions

The design of advanced and versatile filters for 6G communications, significantly immune to manufacturing imperfections, has been introduced in this paper by means of a systematic and rigorous framework. The principal concept is to extract new roots and poles of the filtering function by enforcing its maximum local peaks to remain below a predetermined attenuation threshold due to a specific set of roots/poles deformations. To obtain the necessary filtering functions, two effective and generalized methods (fully independent of the filter’s central frequency), explicitly the SbOM and the CoCPM, have been introduced. According to the SbOM, the roots of the new polynomial are retrieved via a system of equations with the prerequisite that the local maxima of the deformed initial polynomial do not exceed a certain limit, without the need of considering their phase. On the other hand, the CoCPM develops an equivalent polynomial through a compression process in the amplitude and frequency regime along with a corresponding threshold concerning the local maxima. For the optimum profile of the featured filtering functions, a versatile criterion has been formulated along with a useful procedure for objectively comparing them to their various counterparts from existing approaches. Both techniques are completely universal in their applicability (with regard to frequency spectrum or device geometry) and can handle any filtering function. Furthermore, they are straightforward to implement, efficient in the compensation of fabrication imperfections, fast to deliver accurate results, and frugal in computational resources. Actually, these aspects constitute the key differences of the novel theoretical analysis with those of the related literature and pave the way for future research, such as the consideration of substrate losses. The proposed methodology has been validated via the design of real-world waveguide and microstrip filters, covering the broad range between 2GHz and 65GHz, which indicate its potential to serve as a trustworthy tool for the construction of high-end 6G filters.

## Data Availability

Data are contained within the article.
